# Supplementary material for: Synthesis and structural analysis of dinucleotides containing 2′,3′-trans-bridged nucleic acids with trans-5,6- or 5,7-fused ring skeleton
Source: Commun Chem. 2025 Mar 22;8:87. doi: 10.1038/s42004-025-01486-2 (PMC11929919; doi:10.1038/s42004-025-01486-2)
Supplement: Supplementary file 2 — Supplementary Material [file 42004_2025_1486_MOESM2_ESM.pdf]

Supporting Information

Synthesis and structural analysis of dinucleotides containing 2′,3′-*trans*-bridged nucleic acids with *trans*-5,6- or 5,7-fused ring skeleton

Takashi Osawa[1], Ryota Nakanishi[1], Keito Uda[1], So Muramoto[1], Satoshi Obika[1,2,*]

[1]*Graduate School of Pharmaceutical Science, Osaka University, 1-6 Yamadaoka, Suita, Osaka 565-0871, Japan*

[2]*Institute for Open and Transdisciplinary Research Initiatives, Osaka University, 1-3 Yamadaoka, Suita, Osaka 565-0871, Japan*

* *email: obika@phs.osaka-u.ac.jp*

Contents

1. **^{1}H-NMR, ^{13}C-NMR, COSY, NOESY spectra of new compounds** S2–S38

2. ***Ab initio* calculation data (compounds 23a, 24a, 28a, and 29a)** S39–S46

1. ^{1}H-NMR ^{13}C-NMR, COSY, NOESY spectra of new compounds

^{1}H-NMR (500 MHz, $CDCl_3$) of compound **8**

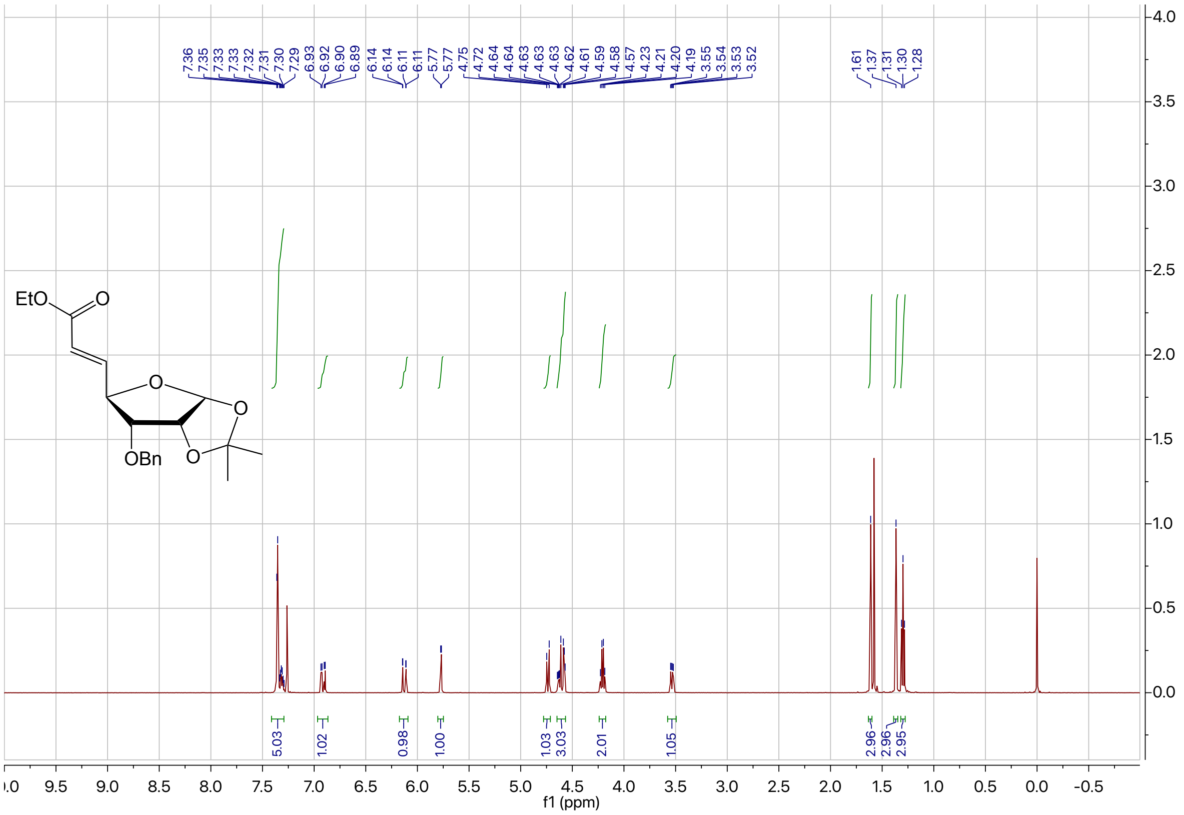

^{13}C-NMR (100 MHz, $CDCl_3$) of compound **8**

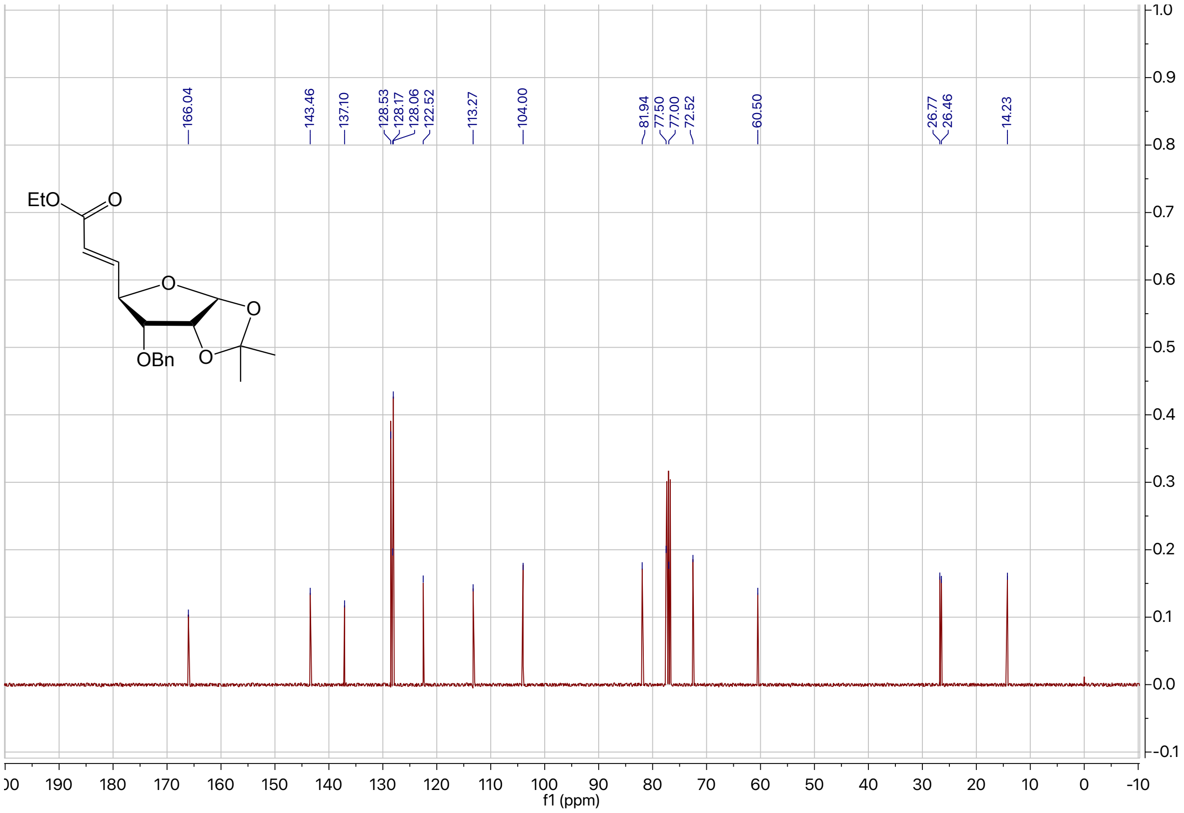

^{1}H-NMR (500 MHz, $CDCl_3$) of compound **9**

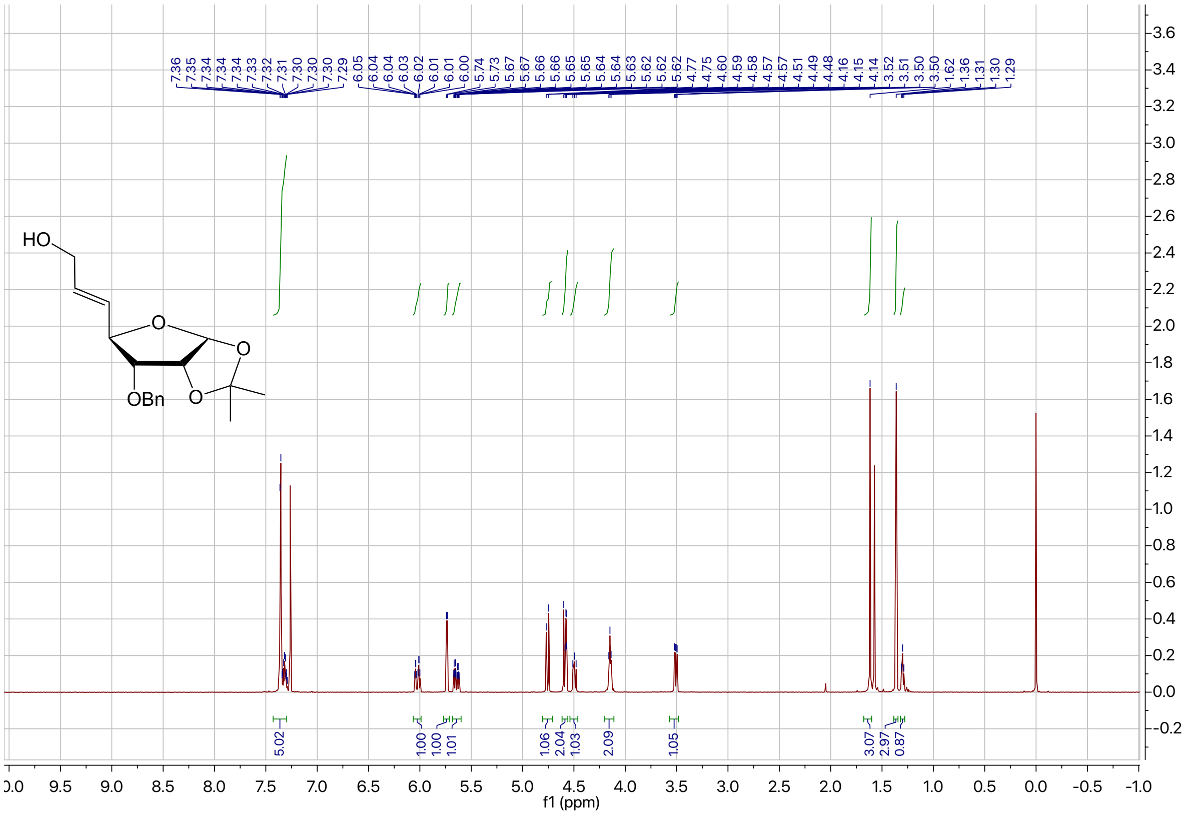

^{13}C-NMR (100 MHz, $CDCl_3$) of compound **9**

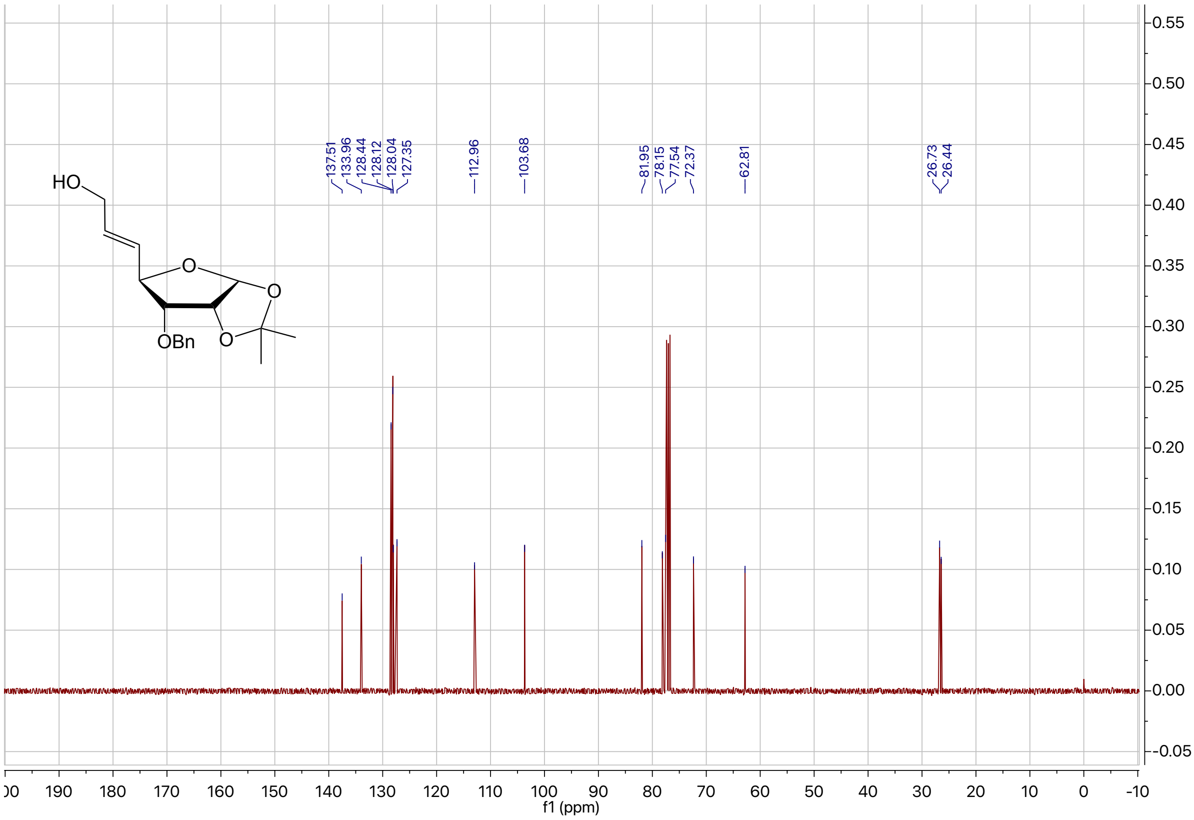

^{1}H-NMR (400 MHz, $CDCl_3$) of compound **4**

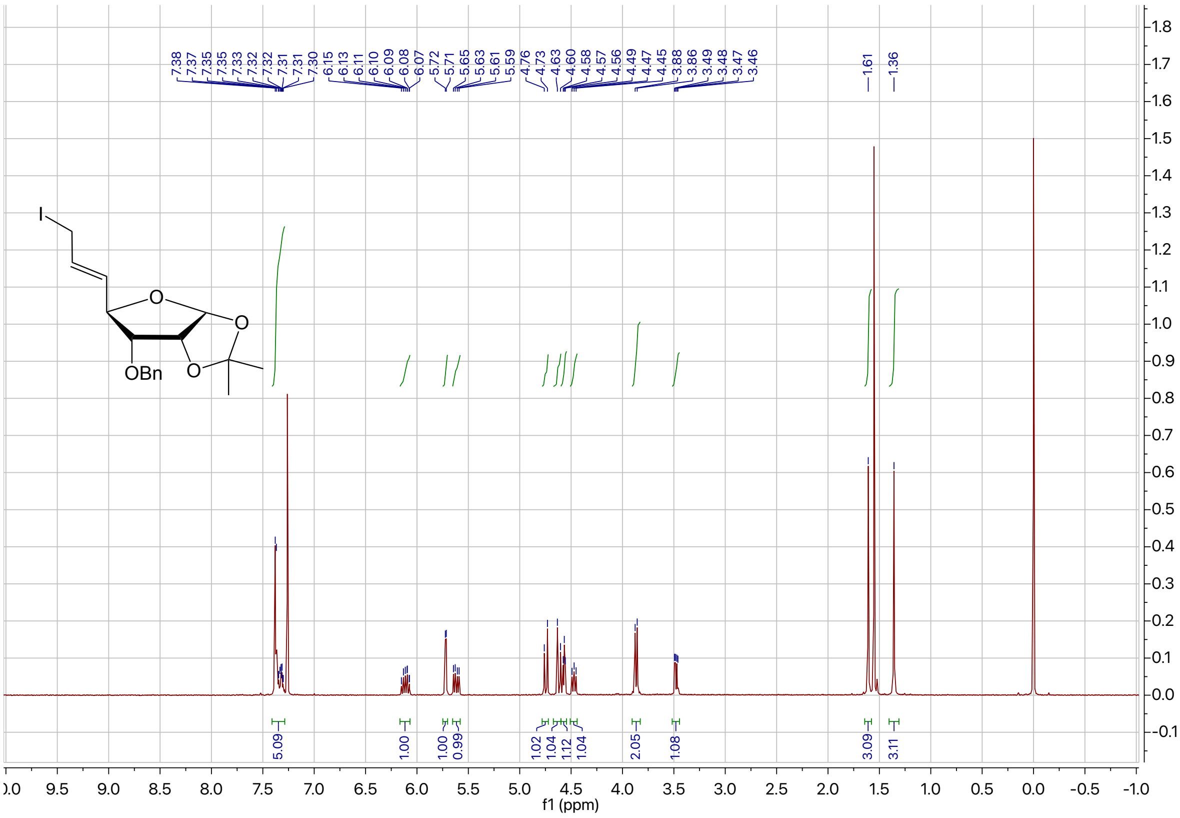

^{13}C-NMR (100 MHz, $CDCl_3$) of compound **4**

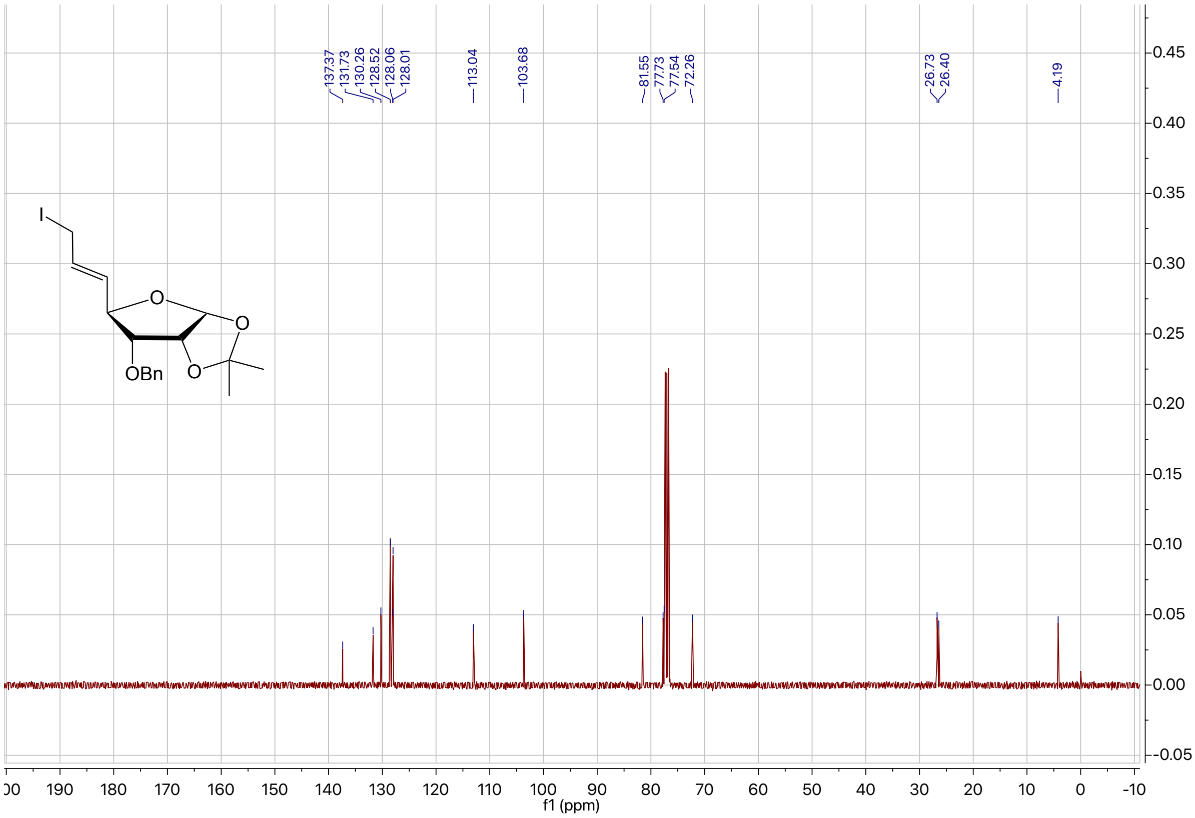

^{1}H-NMR (400 MHz, $CDCl_3$) of compound **12**

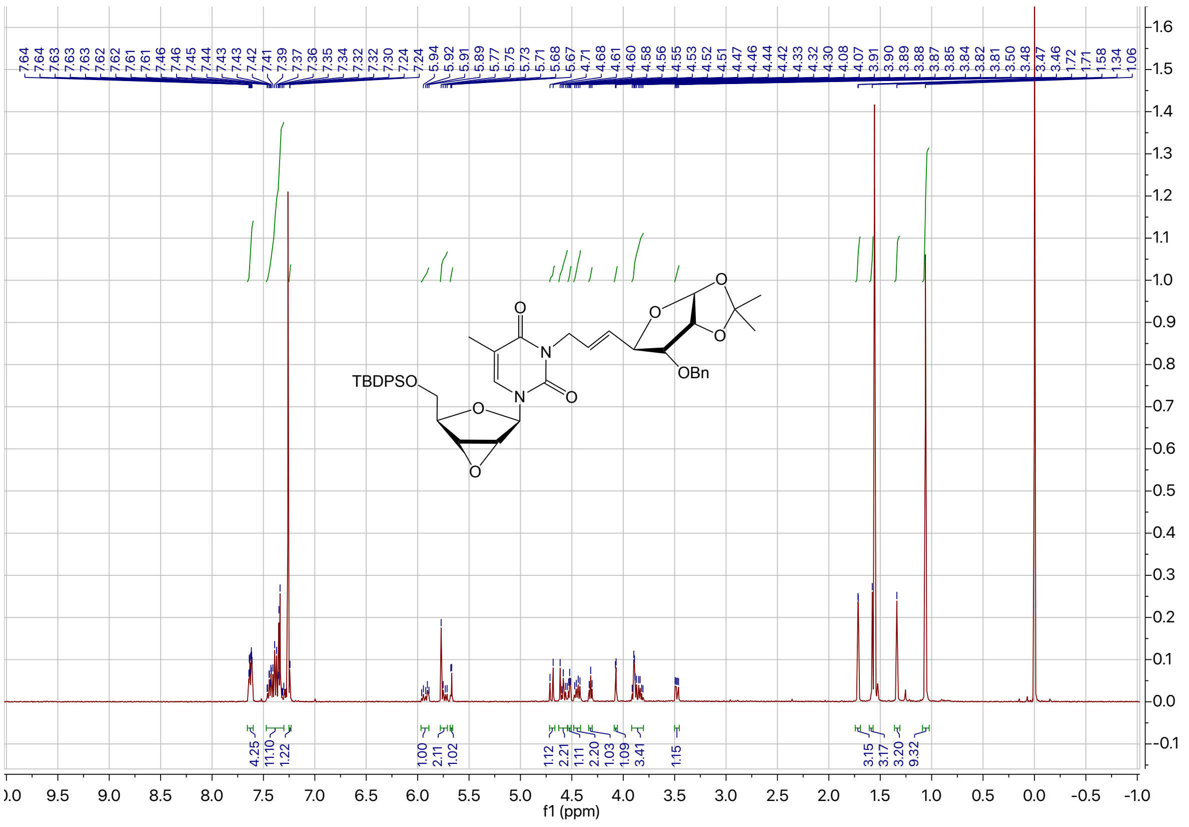

^{13}C-NMR (100.5 MHz, $CDCl_3$) of compound **12**

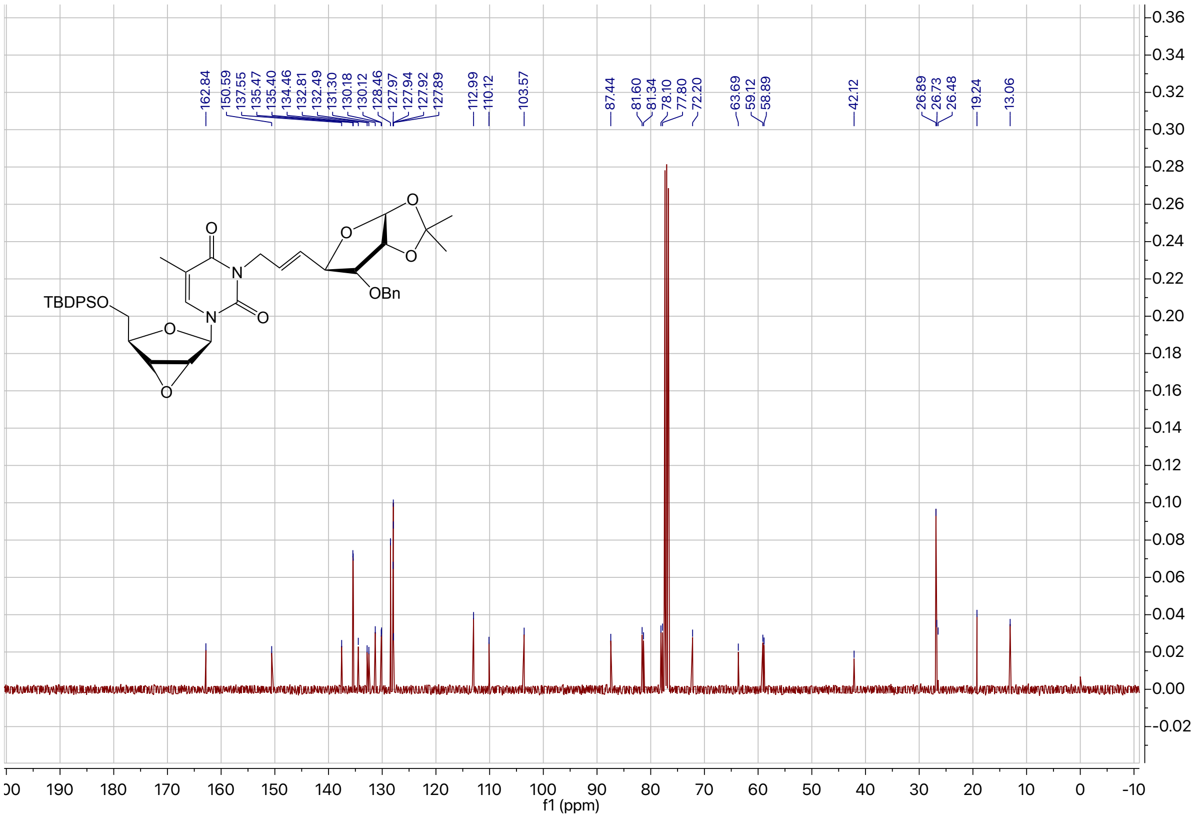

^{1}H-NMR (400 MHz, $CDCl_3$) of compound **10**

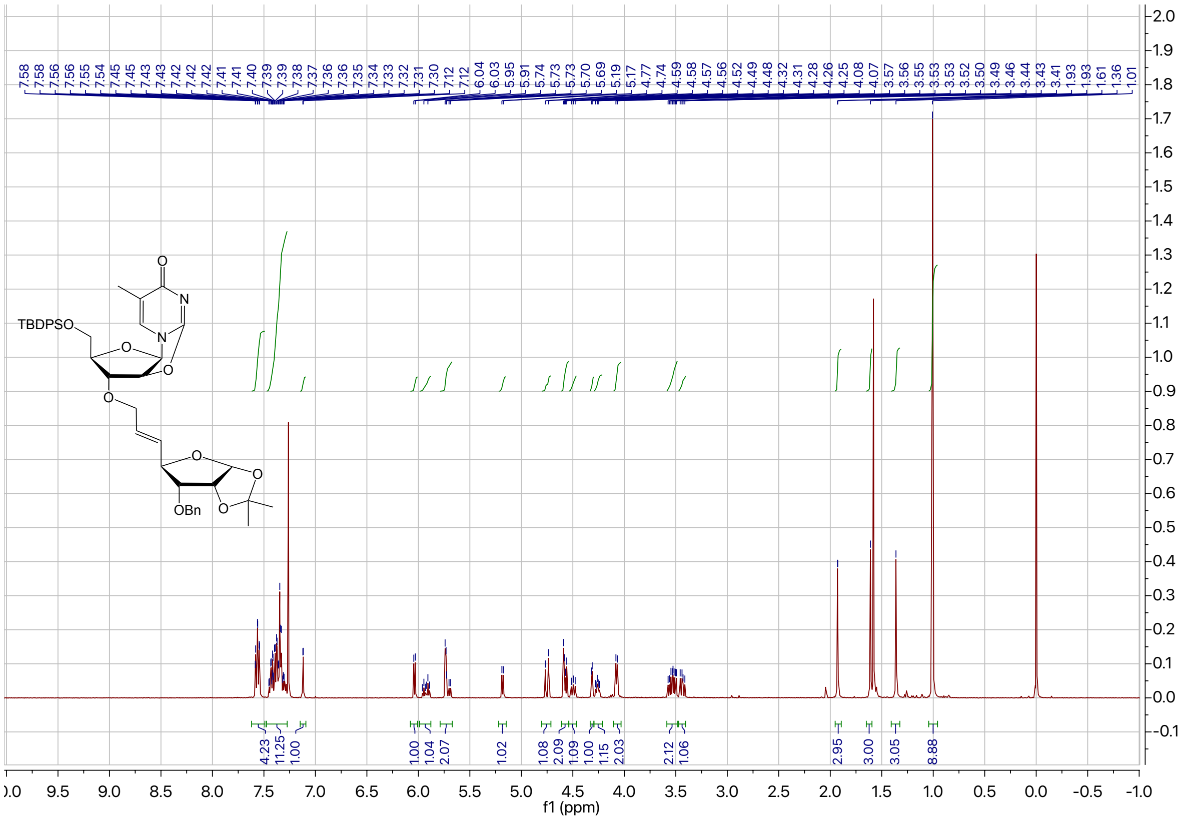

^{13}C-NMR (100 MHz, $CDCl_3$) of compound **10**

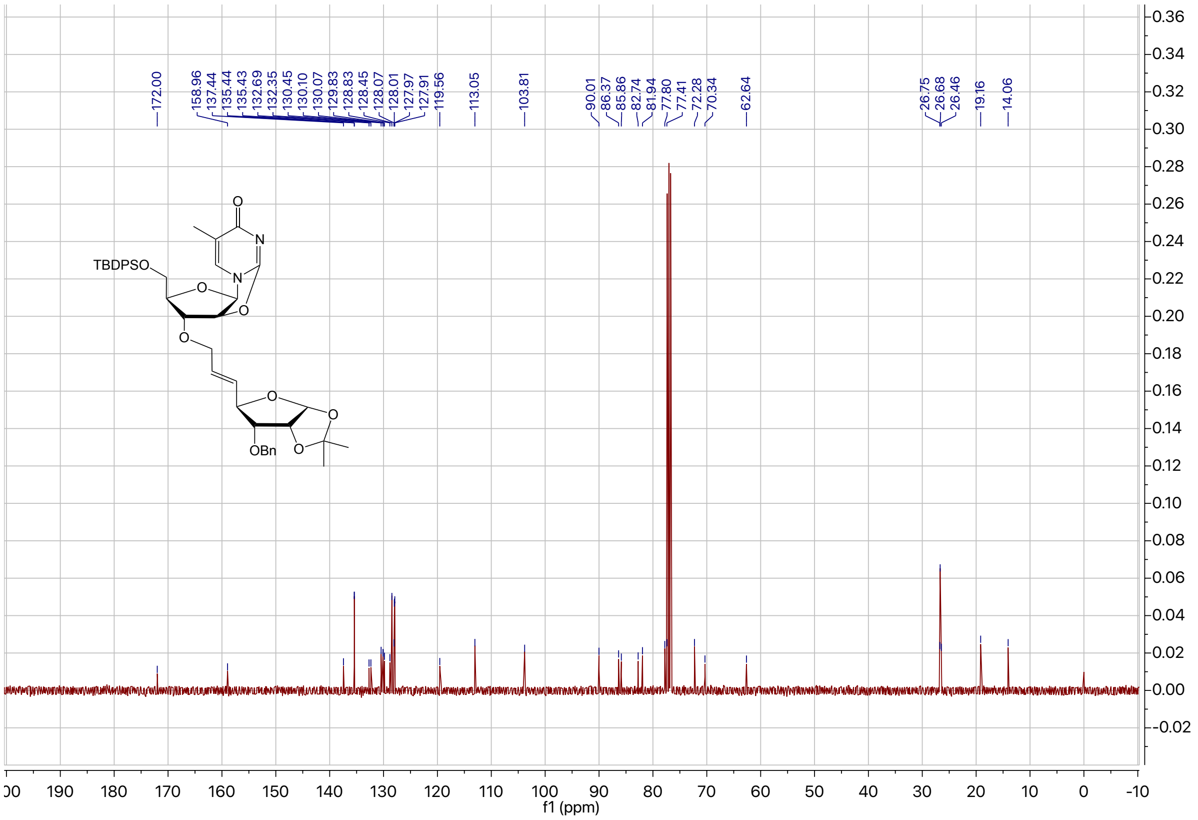

^{1}H-NMR (500 MHz, $CDCl_3$) of compound **1**

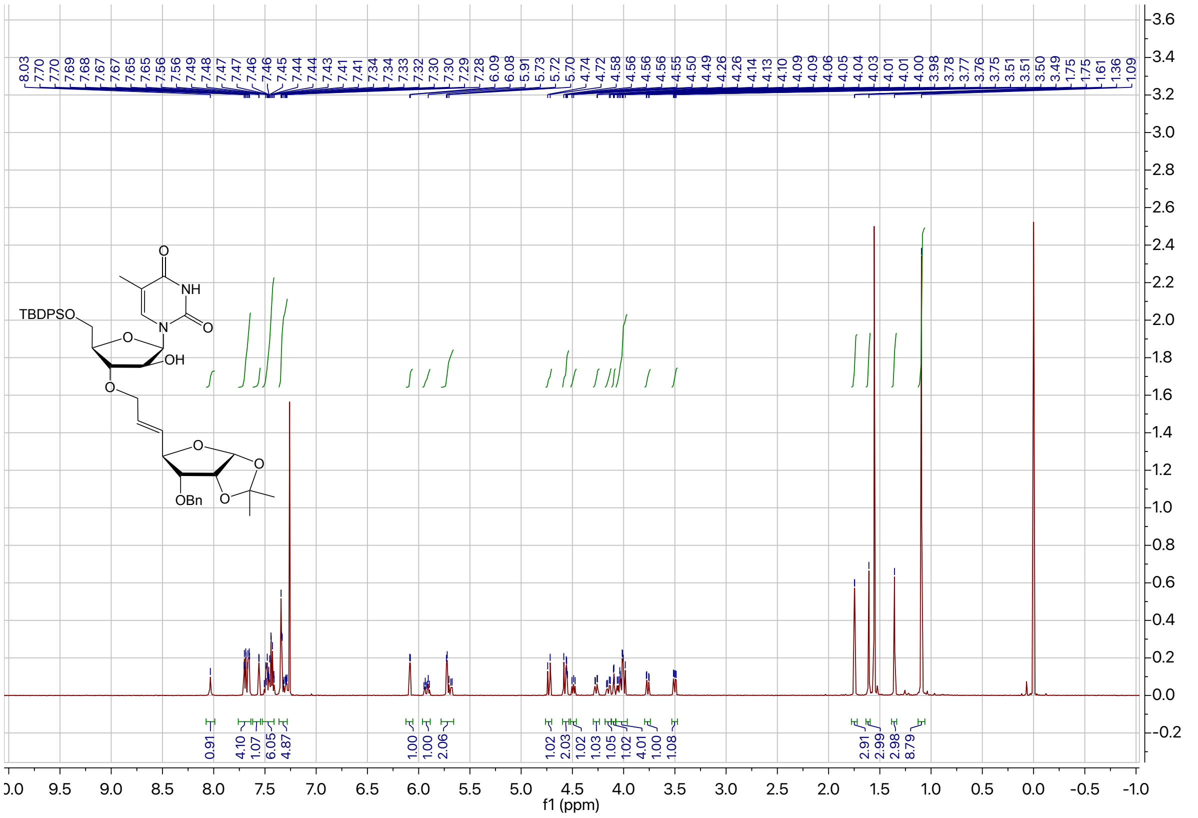

^{13}C-NMR (126 MHz, $CDCl_3$) of compound **1**

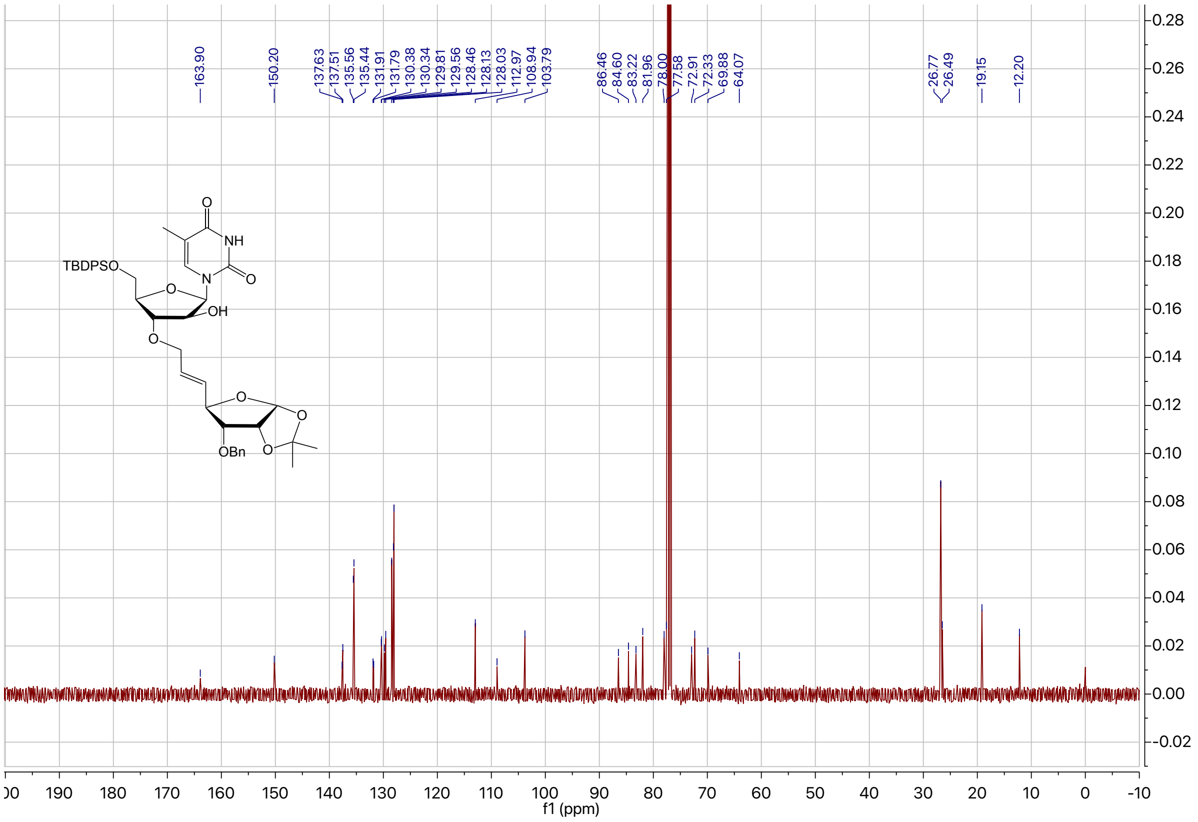

^{1}H-NMR (500 MHz, $CDCl_3$) of compound **11**

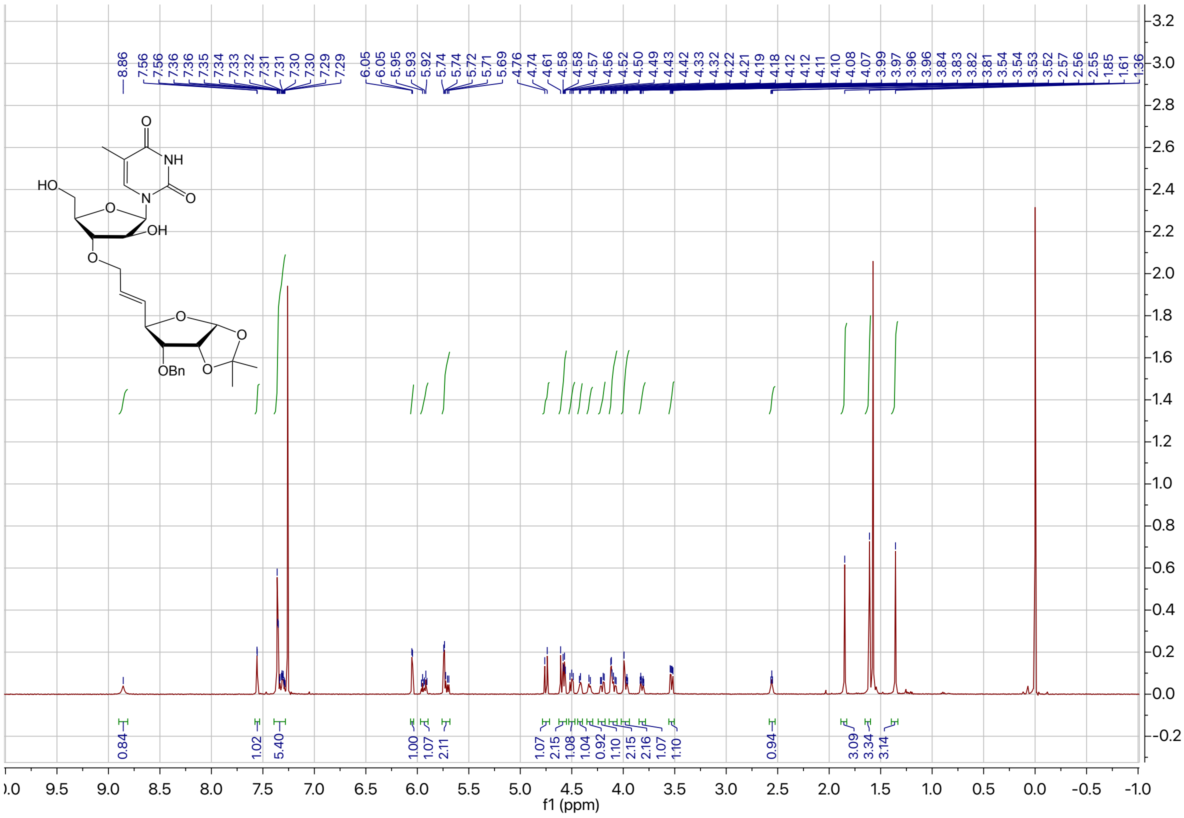

^{13}C-NMR (126 MHz, $CDCl_3$) of compound **11**

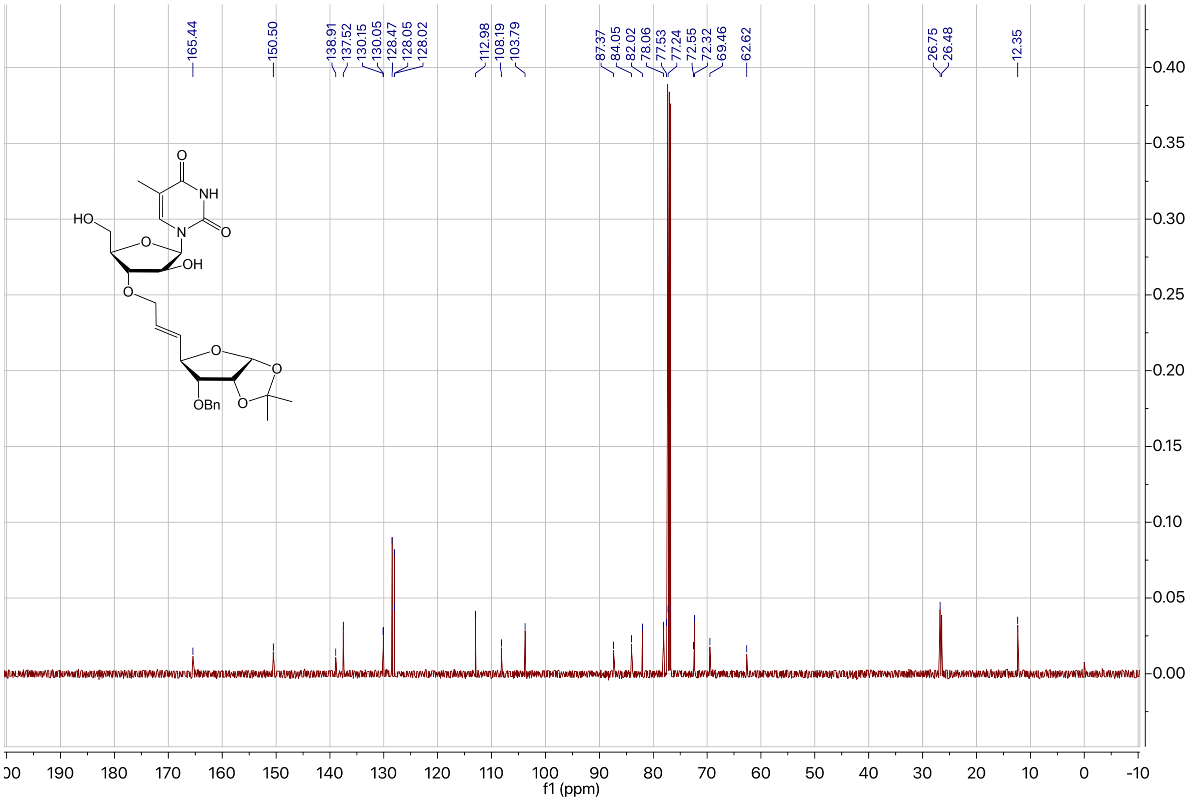

^{1}H-NMR (400 MHz, $CDCl_3$) of compound **13**

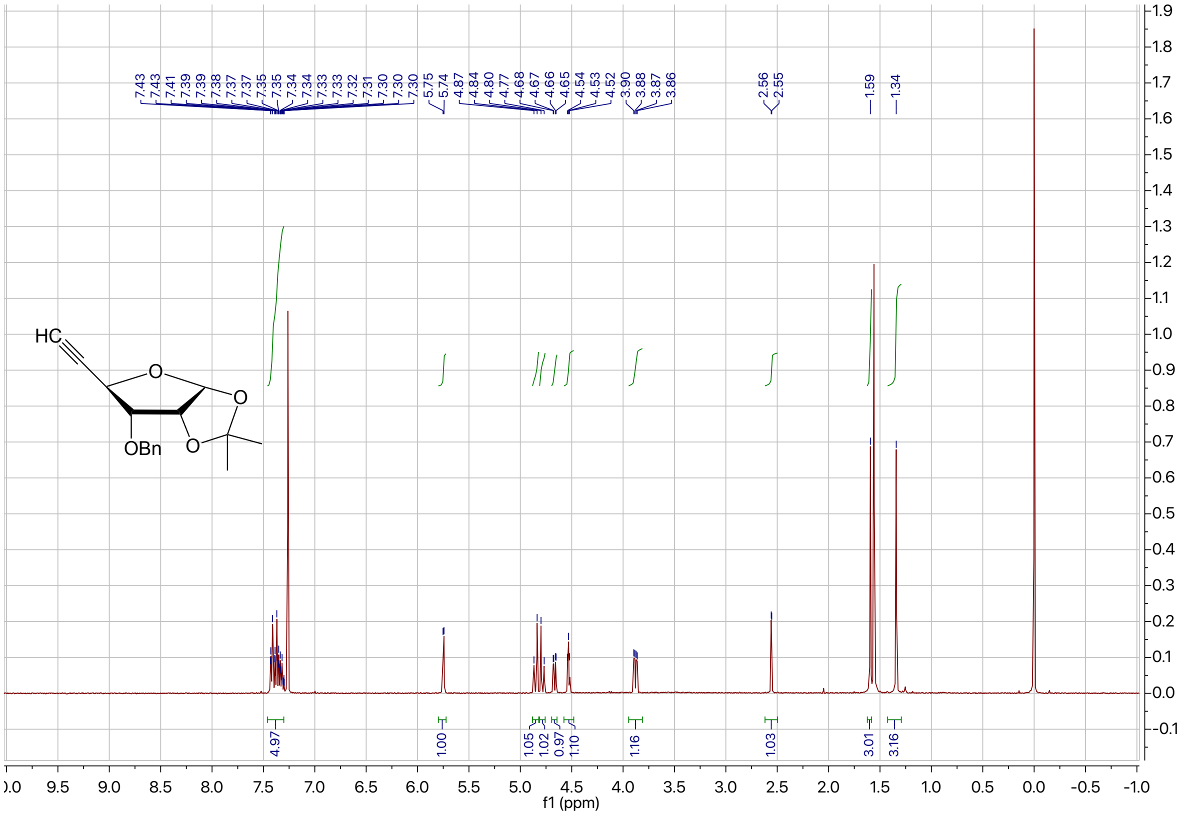

^{13}C-NMR (100 MHz, $CDCl_3$) of compound **13**

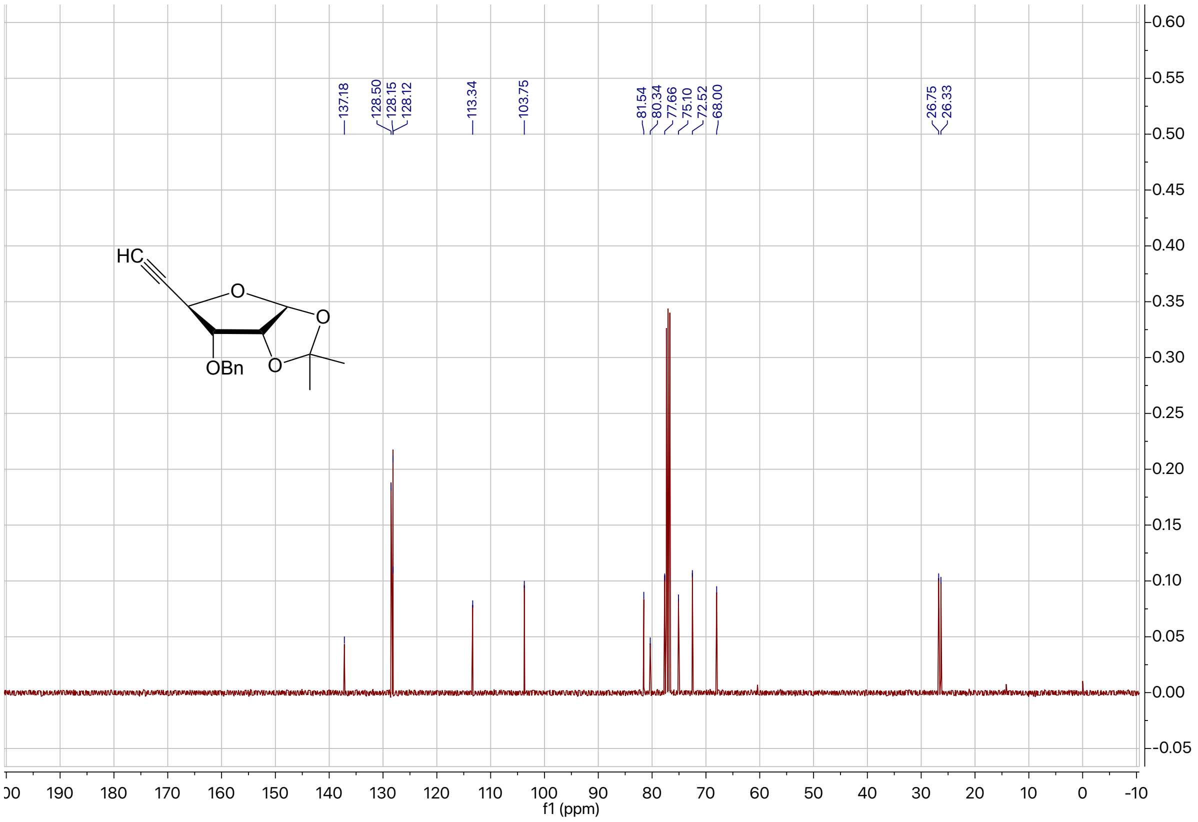

^{1}H-NMR (400 MHz, $CDCl_3$) of compound **14**

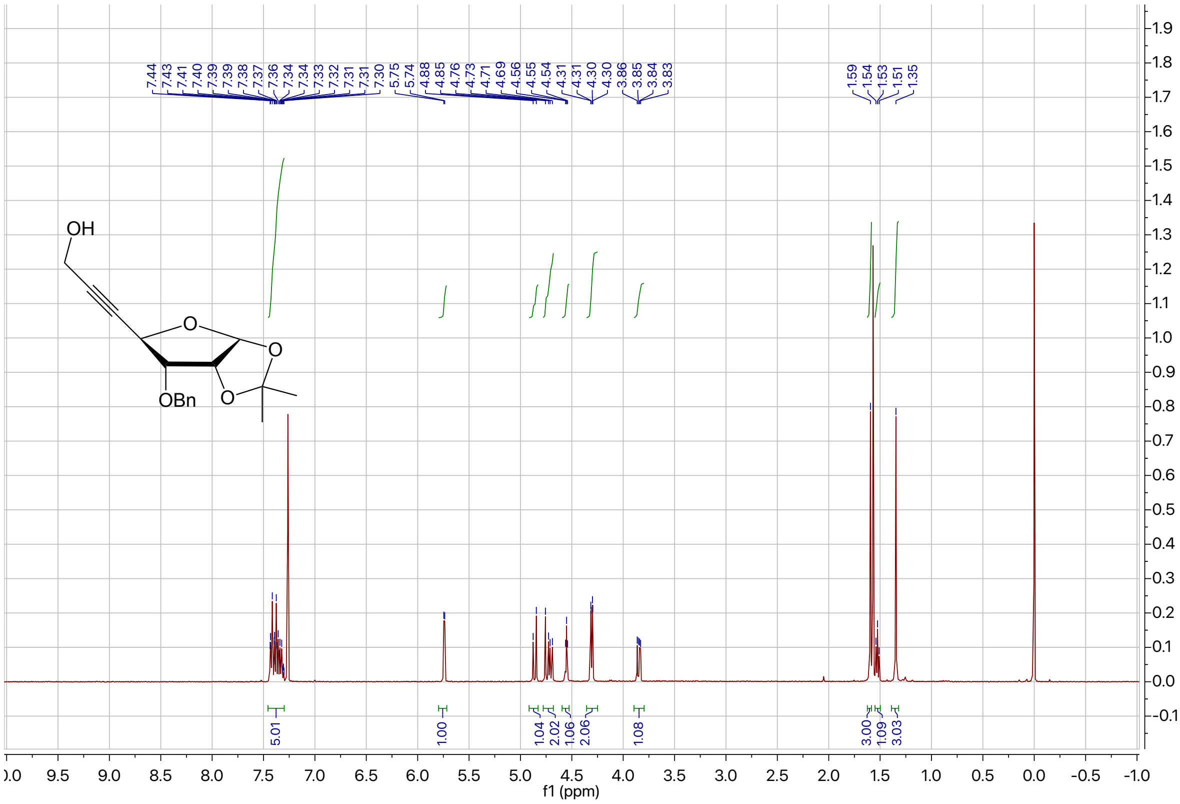

^{13}C-NMR (126 MHz, $CDCl_3$) of compound **14**

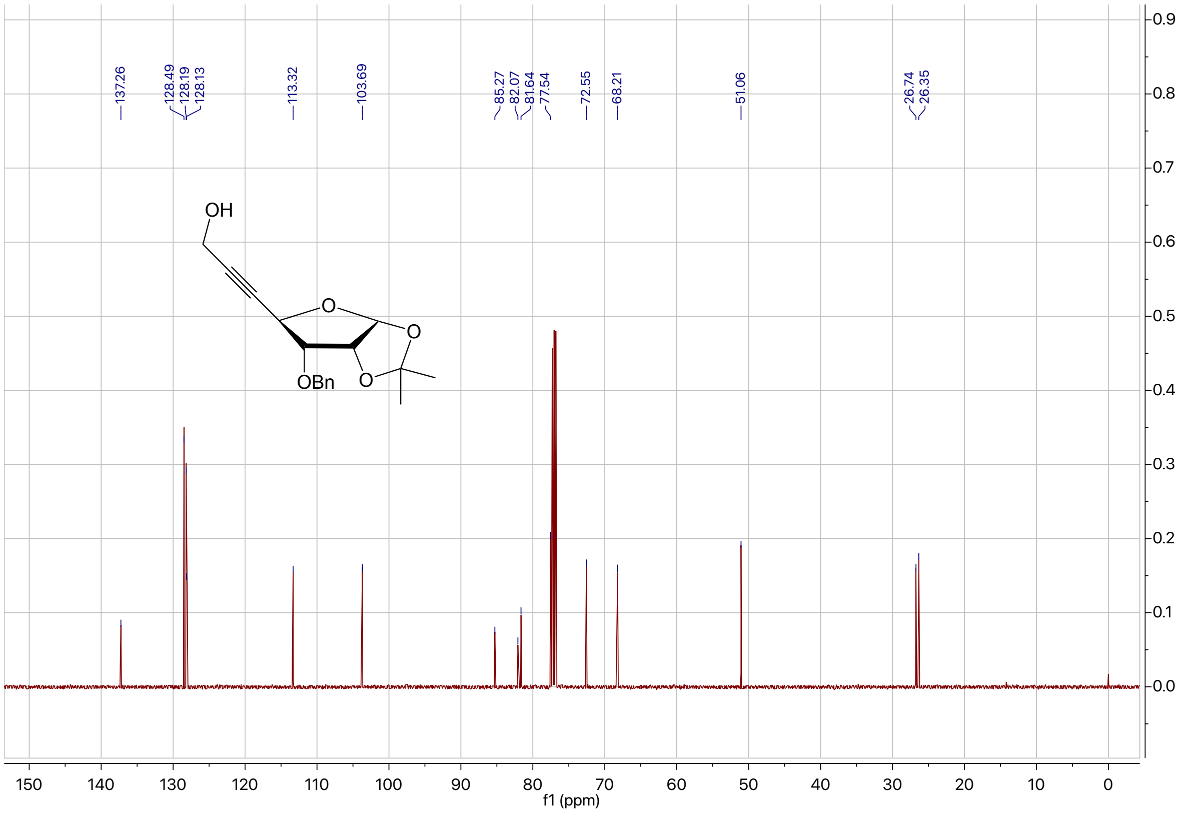

^{1}H-NMR (300 MHz, $CDCl_3$) of compound **5**

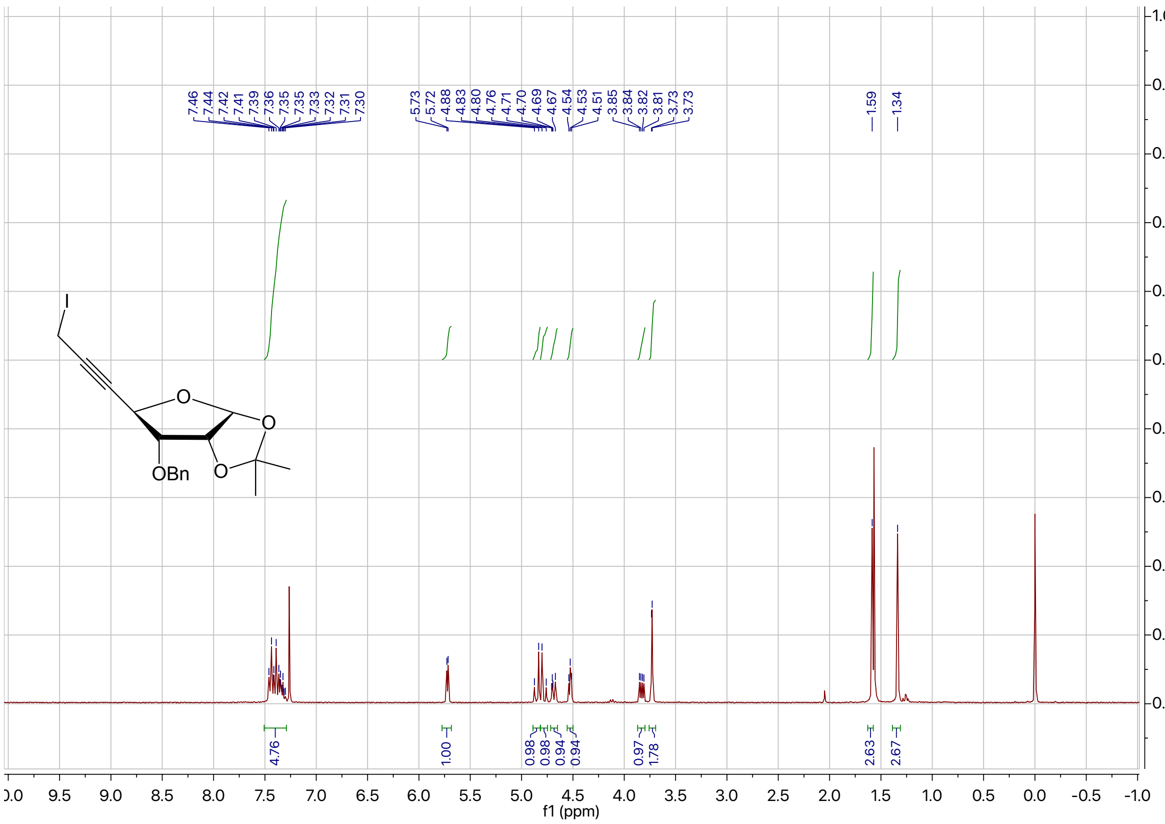

^{13}C-NMR (126 MHz, $CDCl_3$) of compound **5**

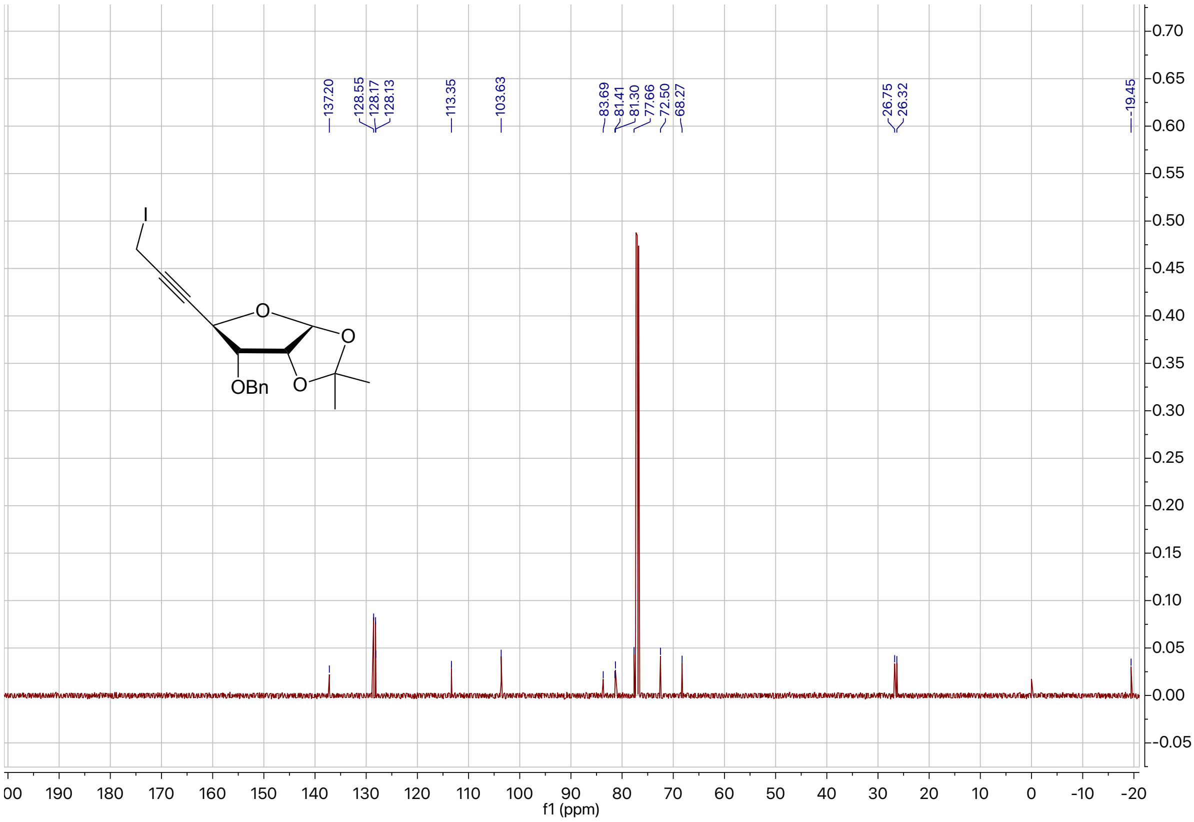

^{1}H-NMR (500 MHz, $CDCl_3$) of compound **15**

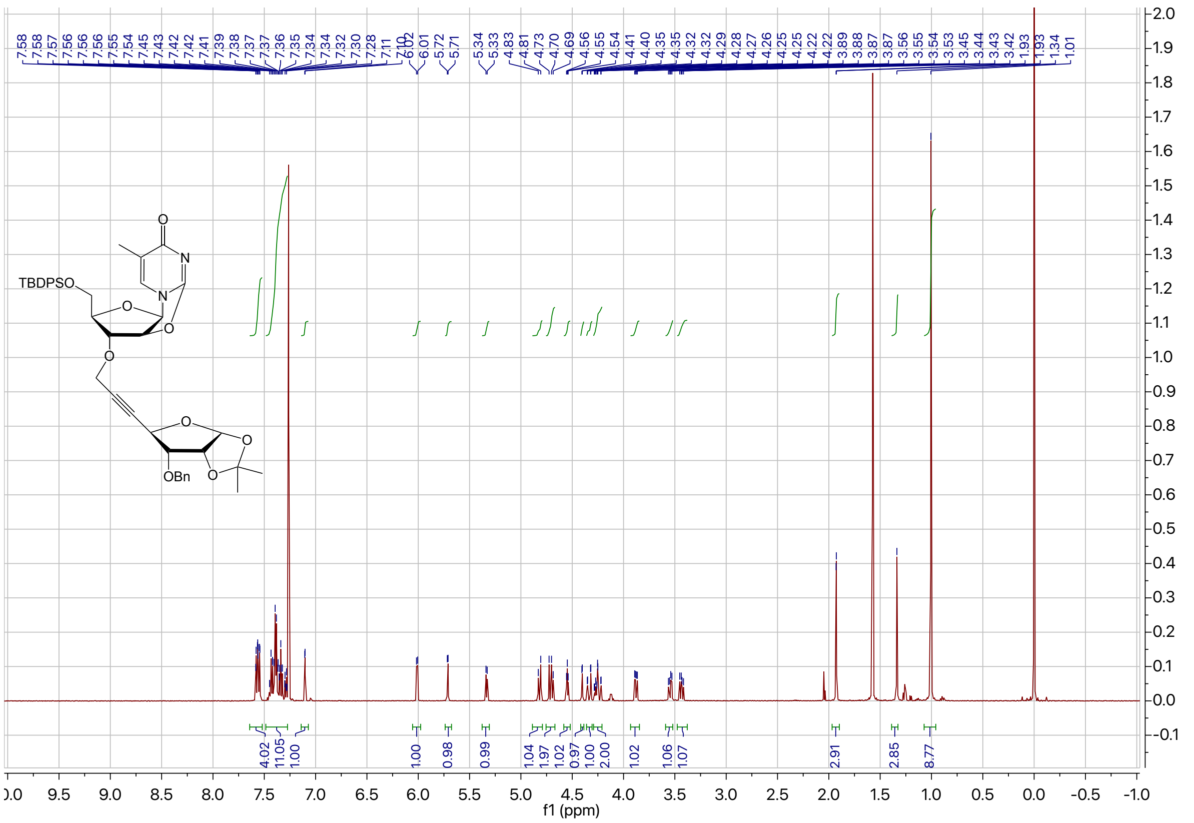

^{13}C-NMR (100 MHz, $CDCl_3$) of compound **15**

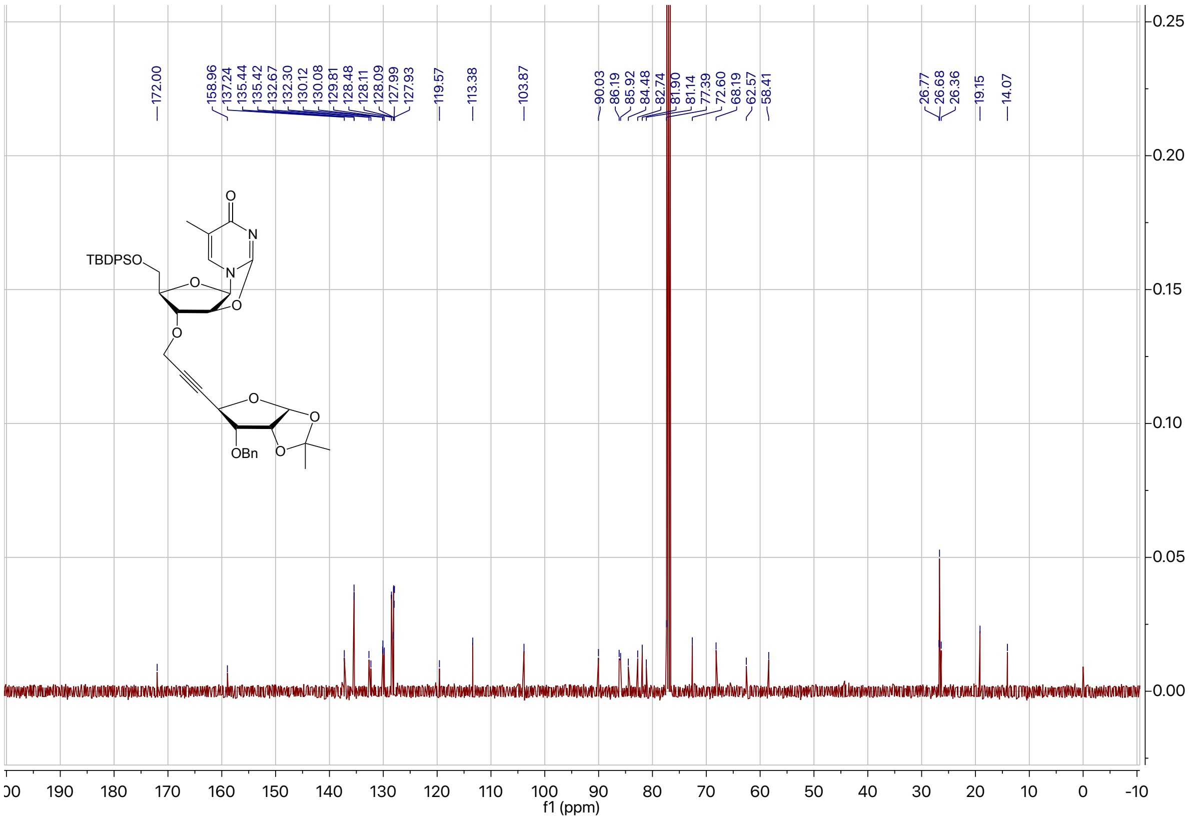

^{1}H-NMR (500 MHz, $CDCl_3$) of compound **2**

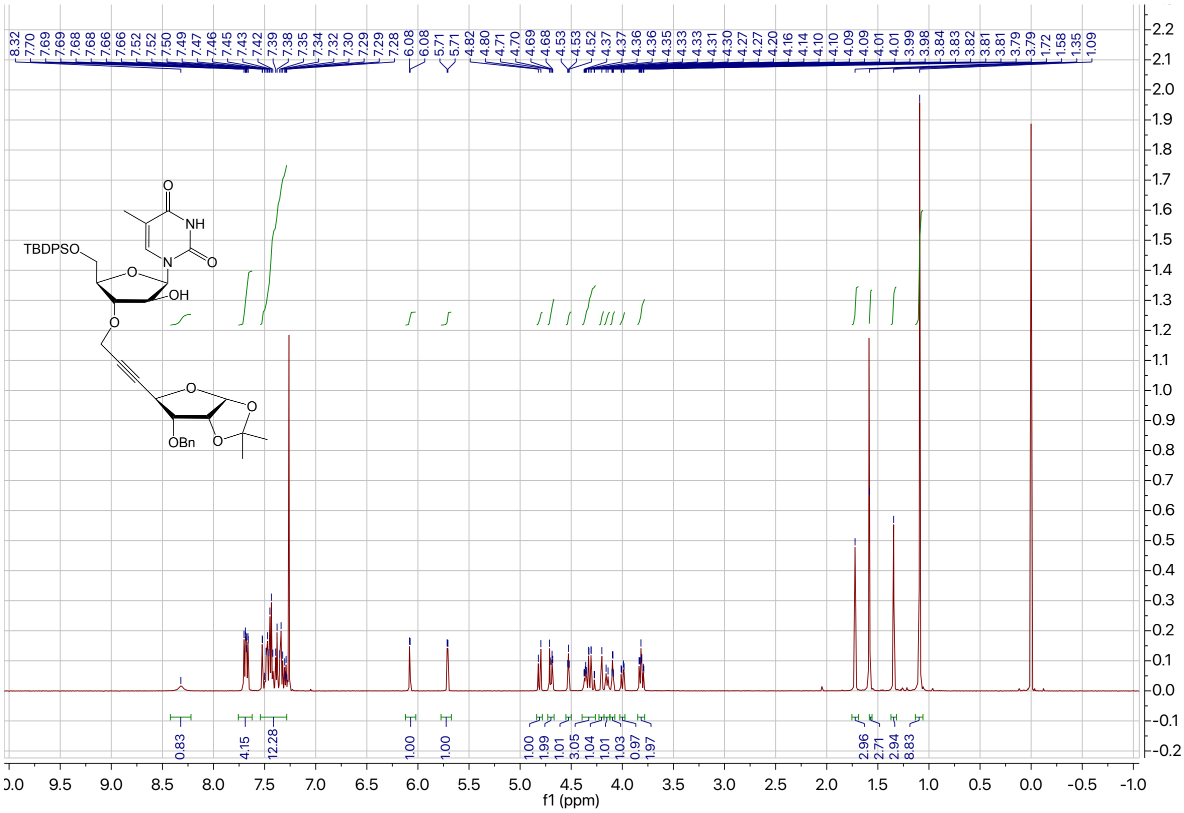

^{13}C-NMR (126 MHz, $CDCl_3$) of compound **2**

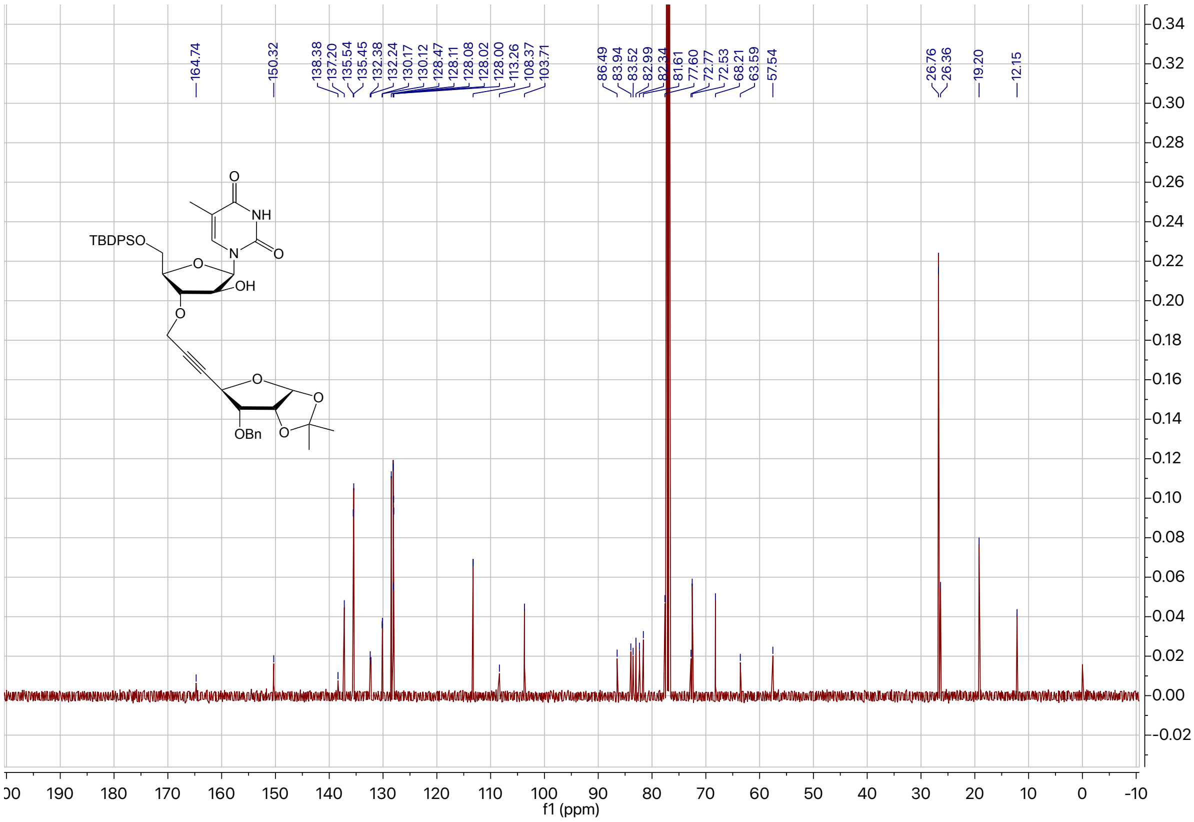

^{1}H-NMR (300 MHz, $CDCl_3$) of compound **16**

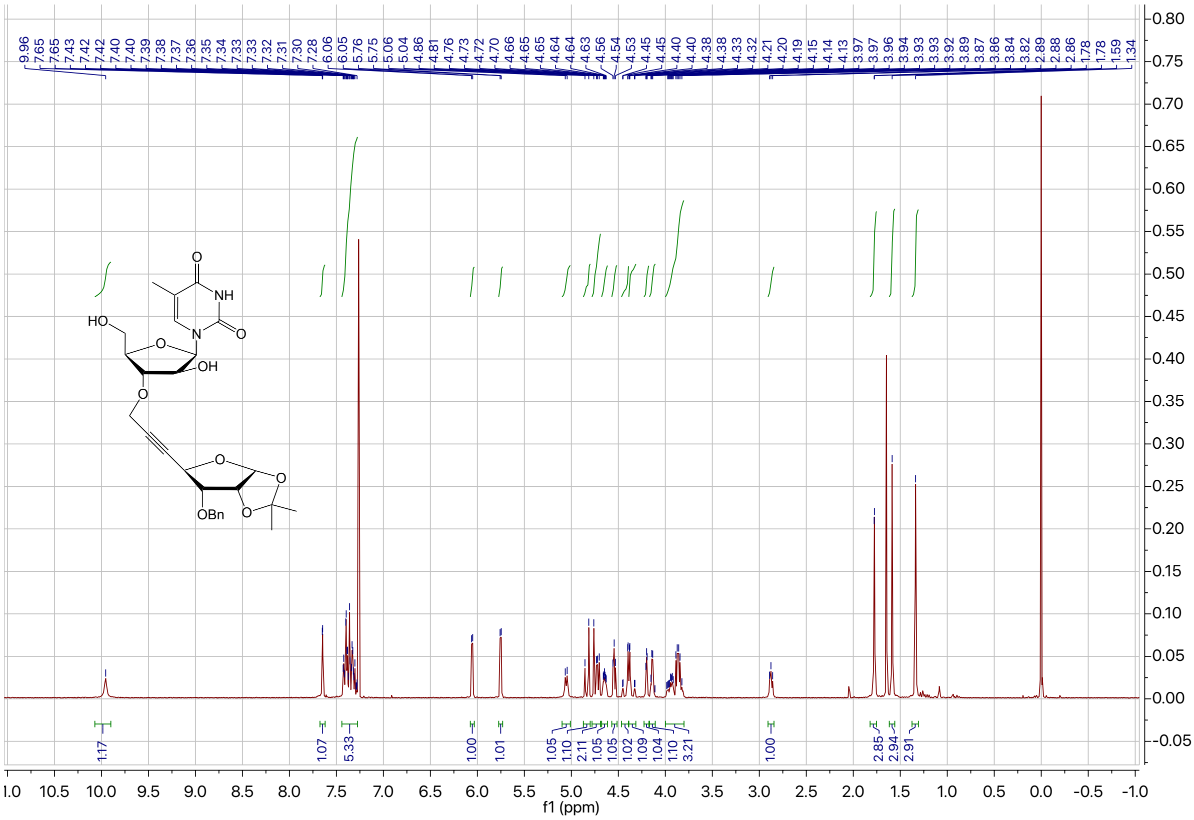

^{13}C-NMR (126 MHz, $CDCl_3$) of compound **16**

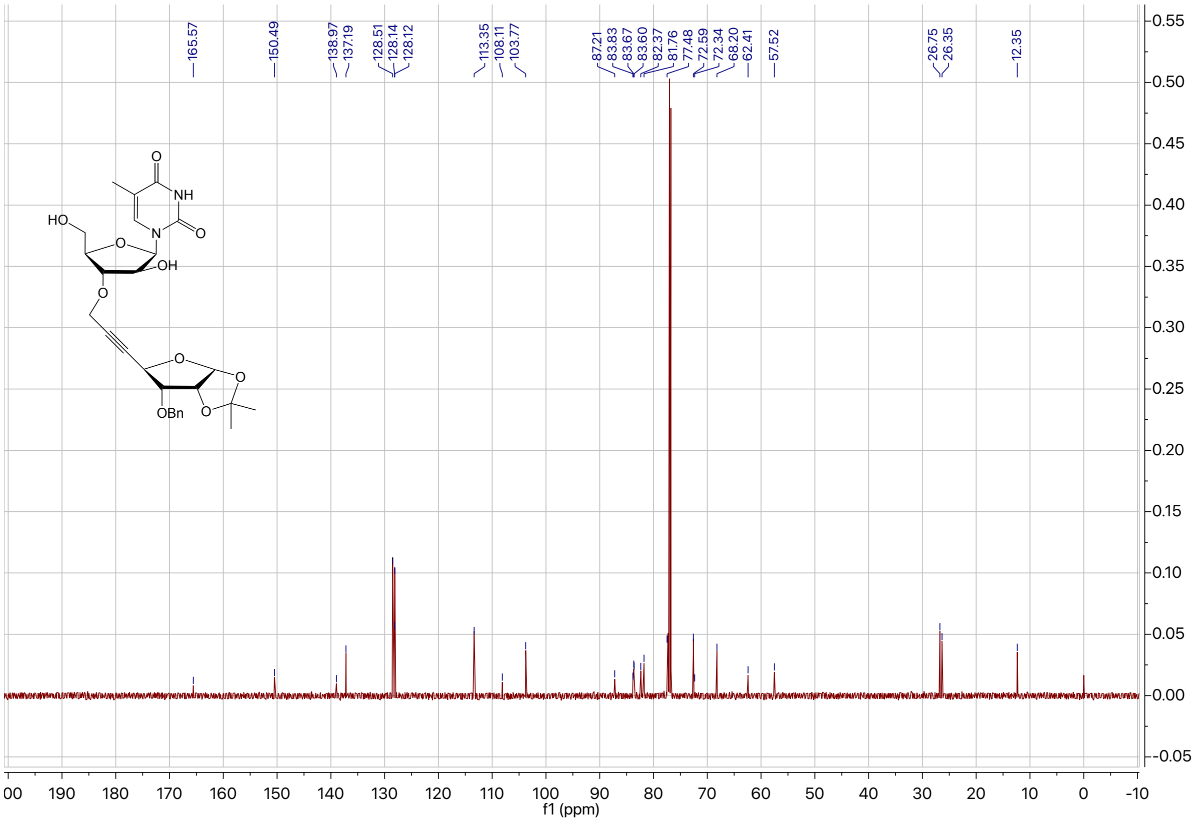

^{1}H-NMR (500 MHz, $CDCl_3$) of compound **17**

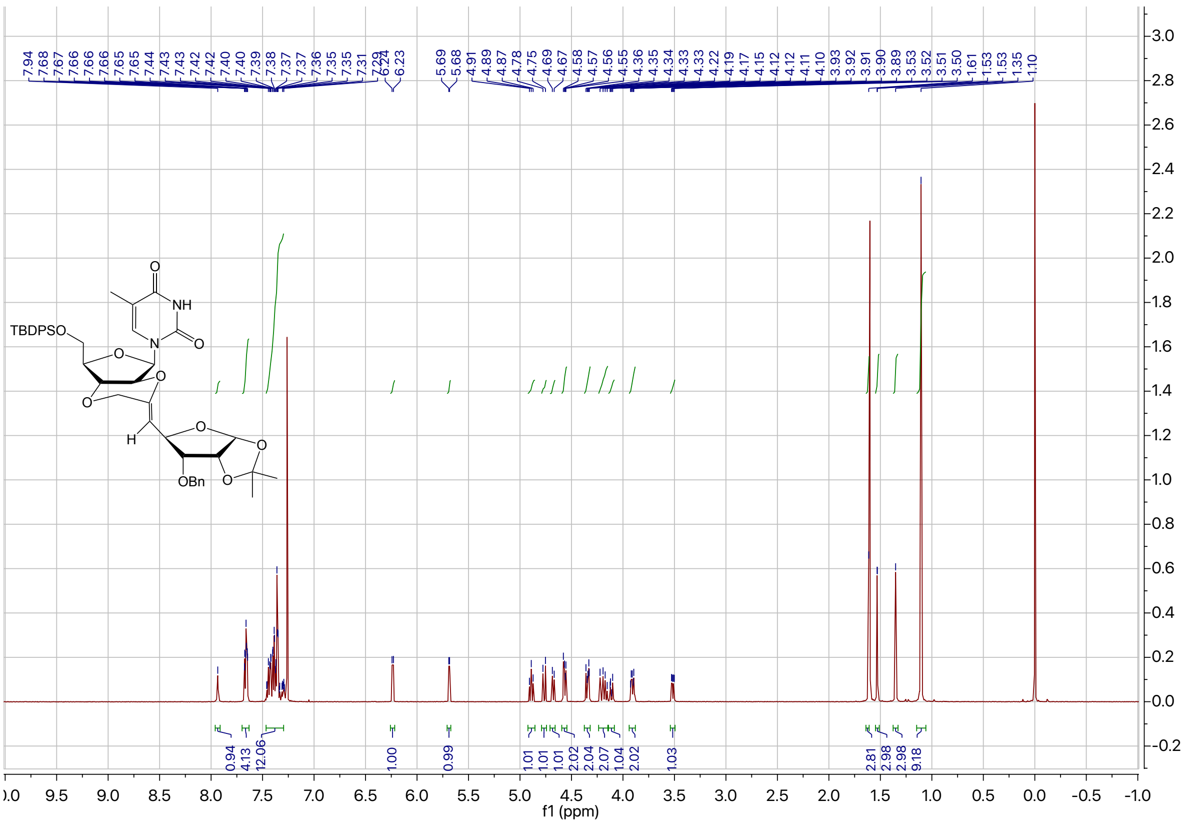

^{13}C-NMR (126 MHz, $CDCl_3$) of compound **17**

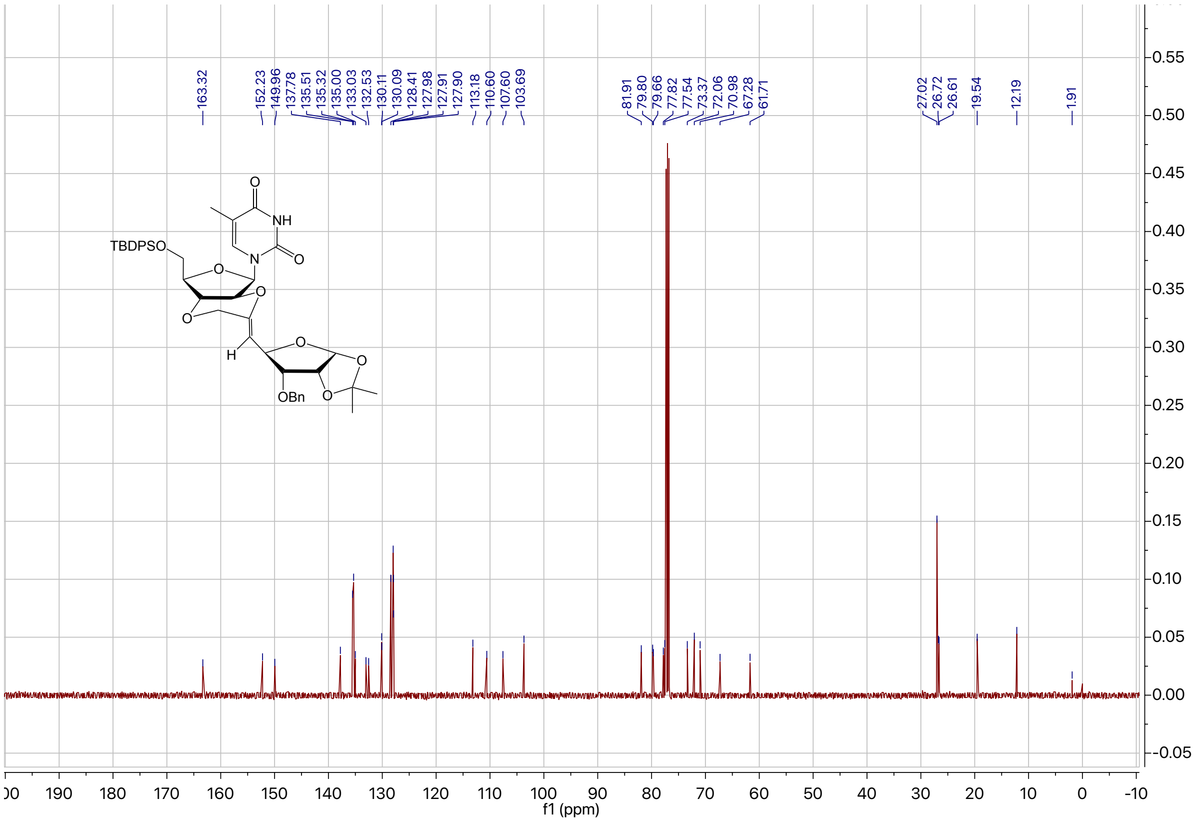

H-H COSY (500 MHz, $CDCl_3$) of compound **17**

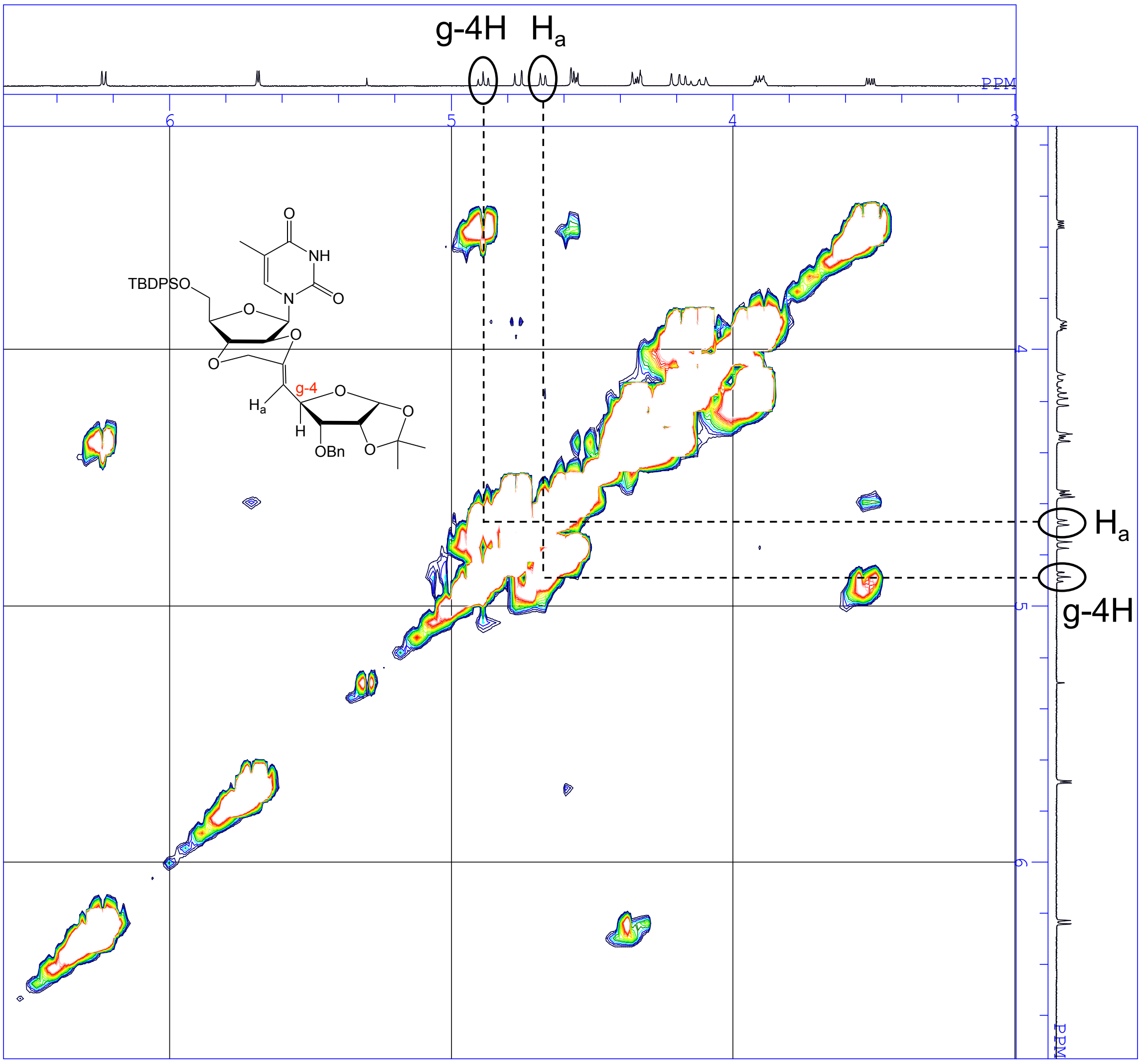

^{1}H-NMR (500 MHz, CD_3CN) of compound **18**

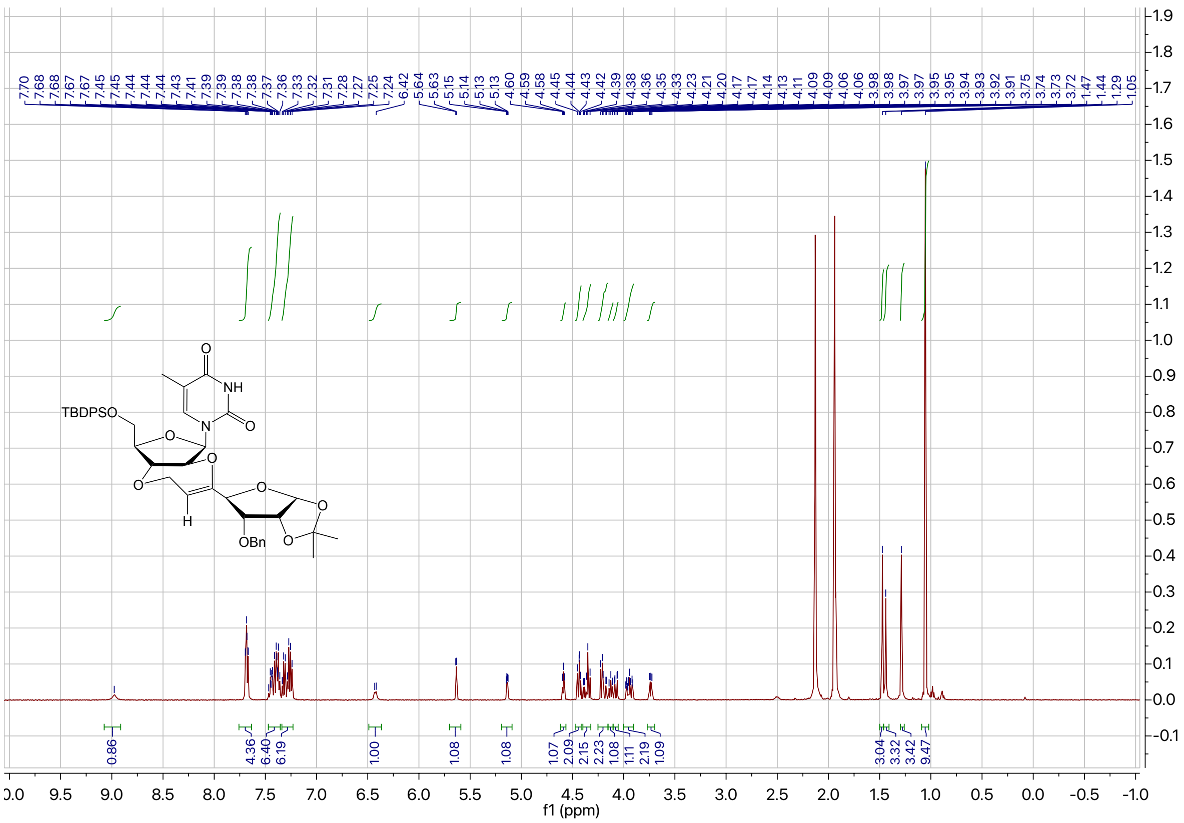

^{13}C-NMR (126 MHz, $CDCl_3$) of compound **18**

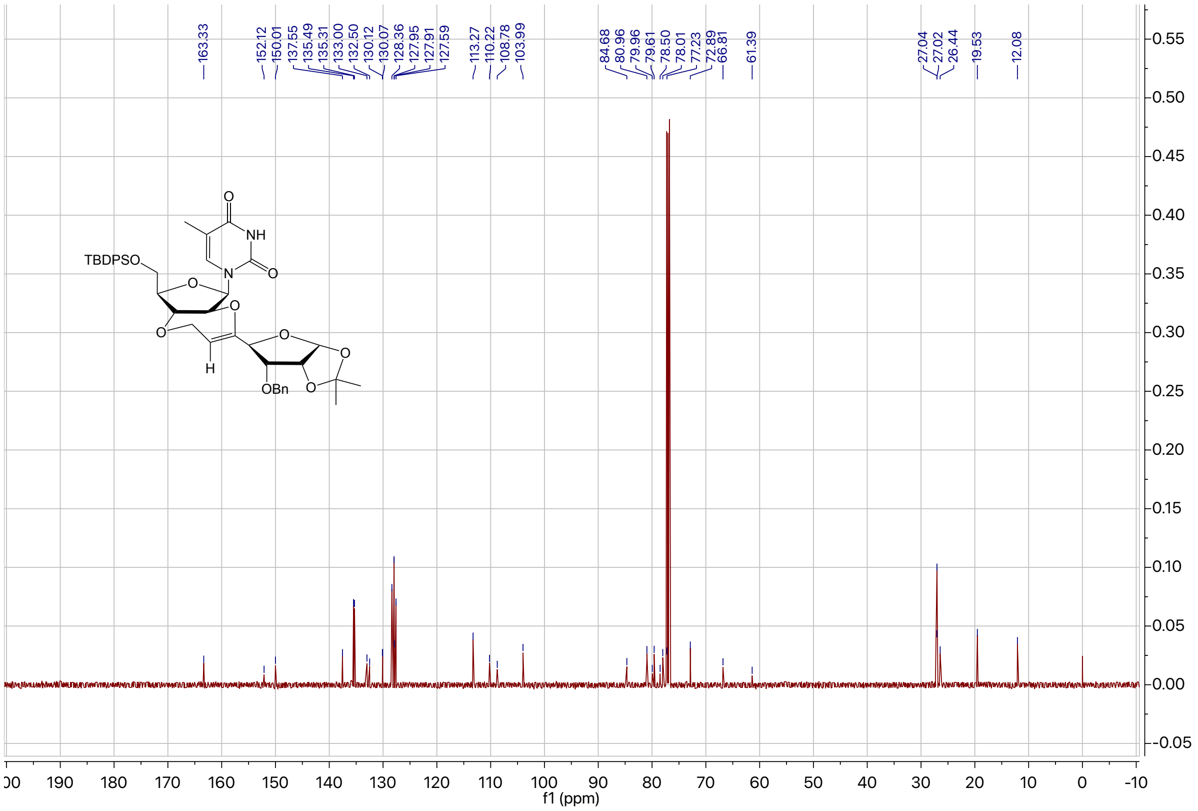

H-H COSY (500 MHz, $CDCl_3$) of compound **18**

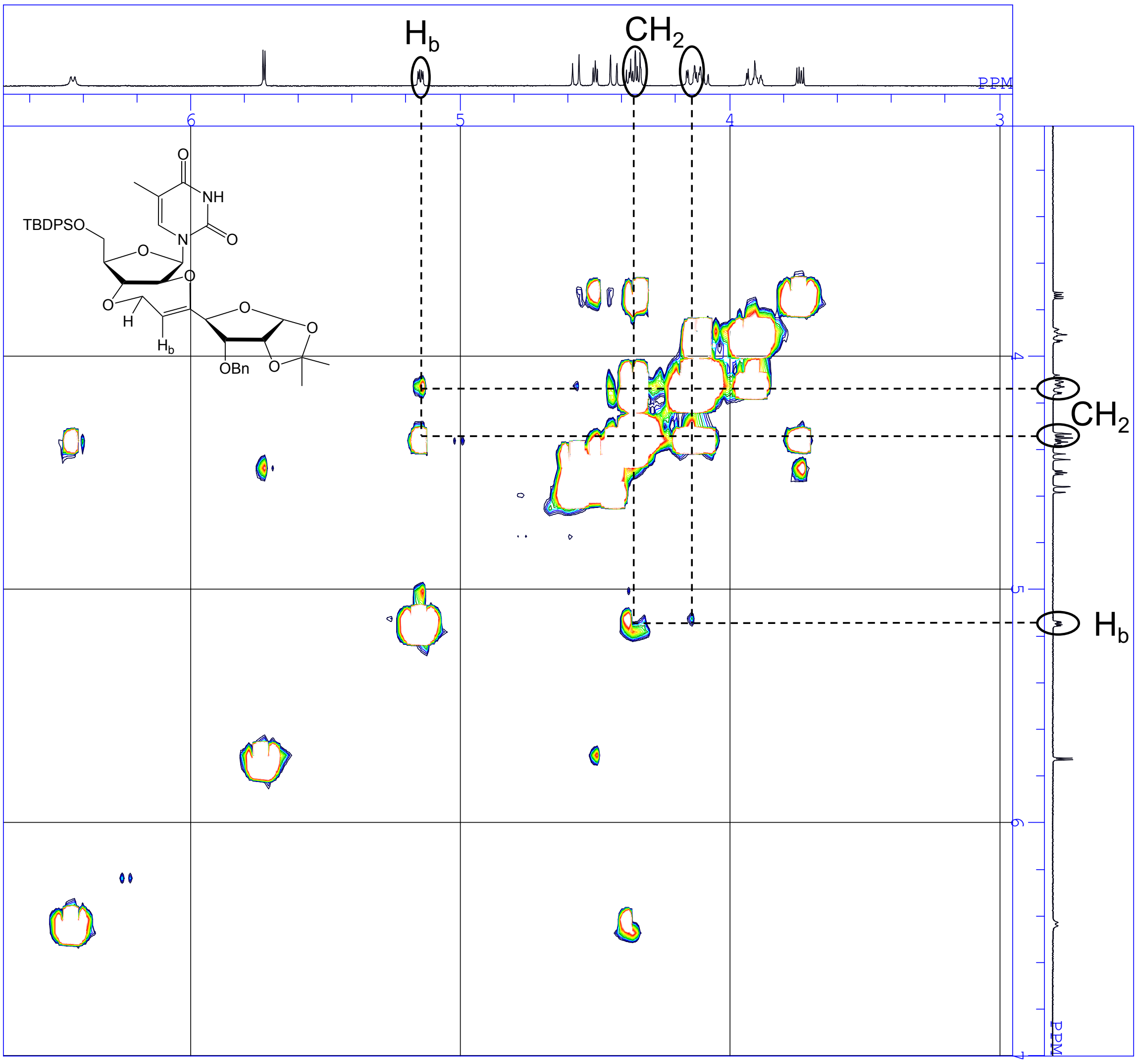

^{1}H-NMR (400 MHz, $CDCl_3$) of compound **19**

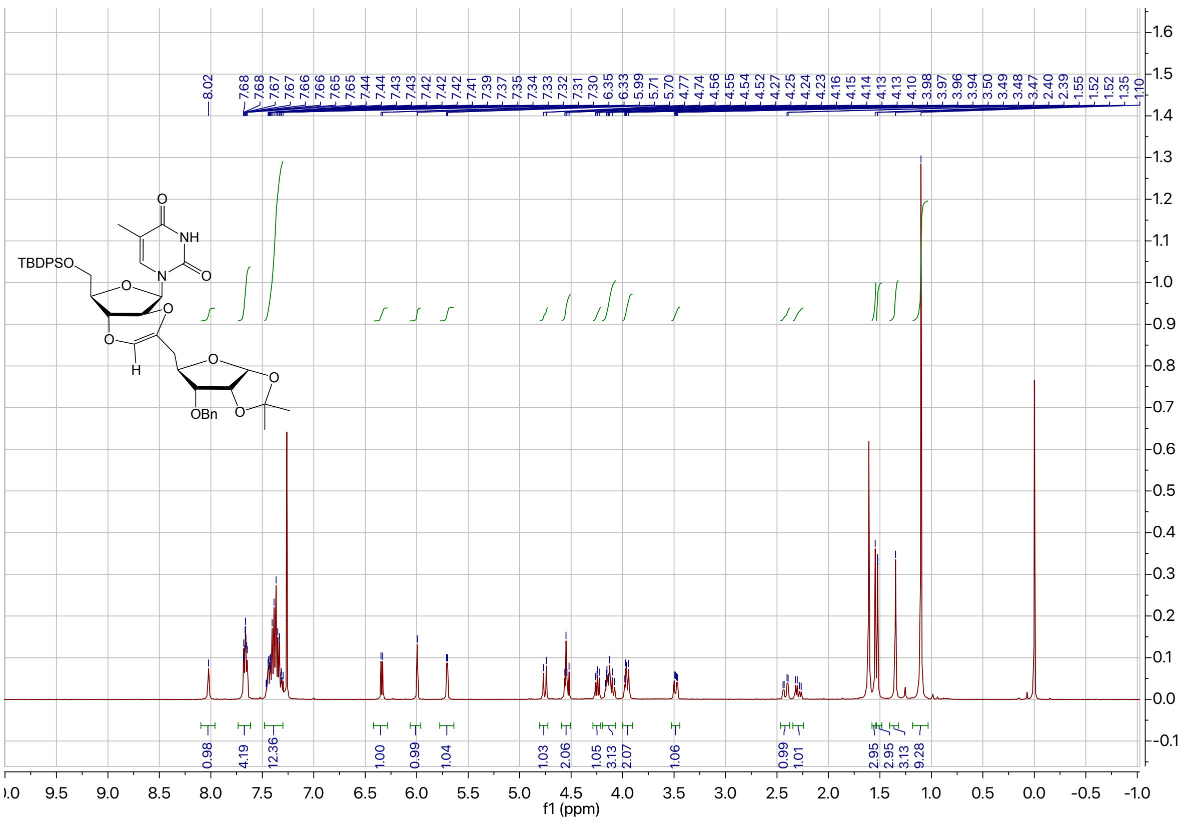

^{13}C-NMR (126 MHz, $CDCl_3$) of compound **19**

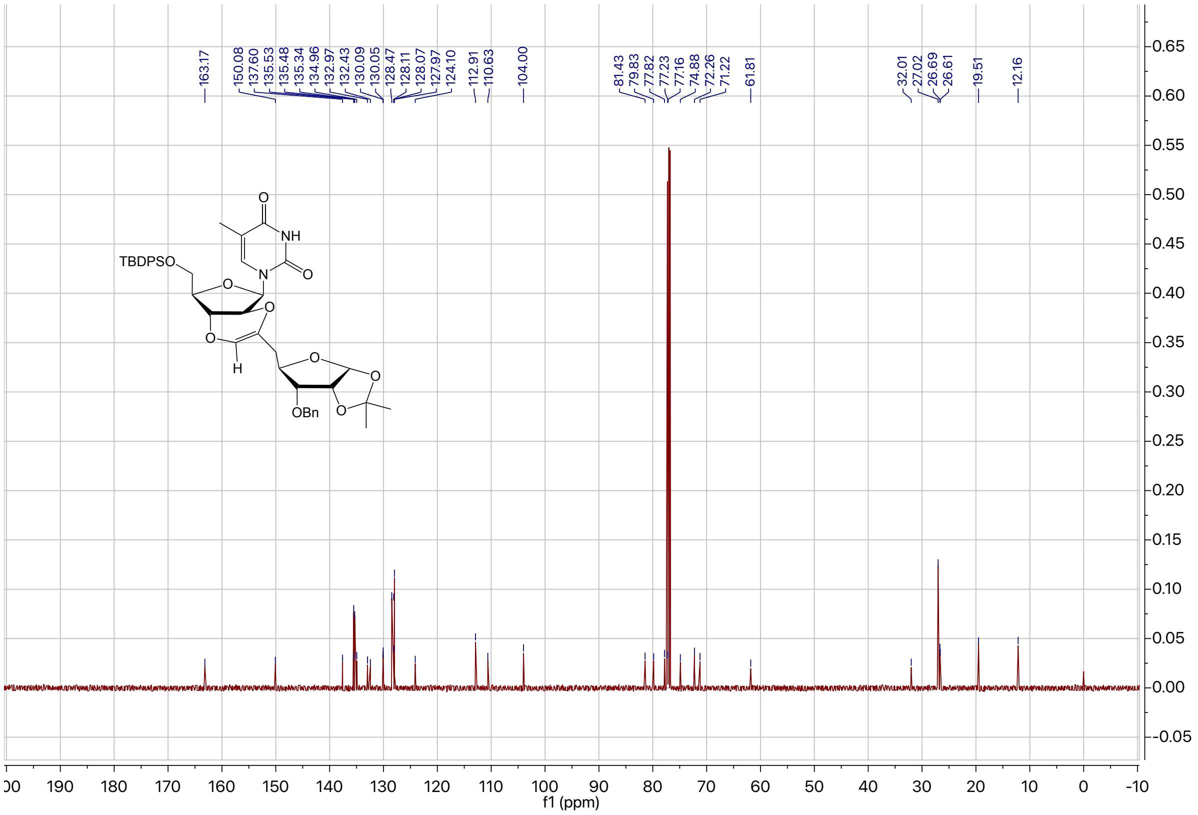

^{1}H-NMR (500 MHz, $CDCl_3$) of compound **20**

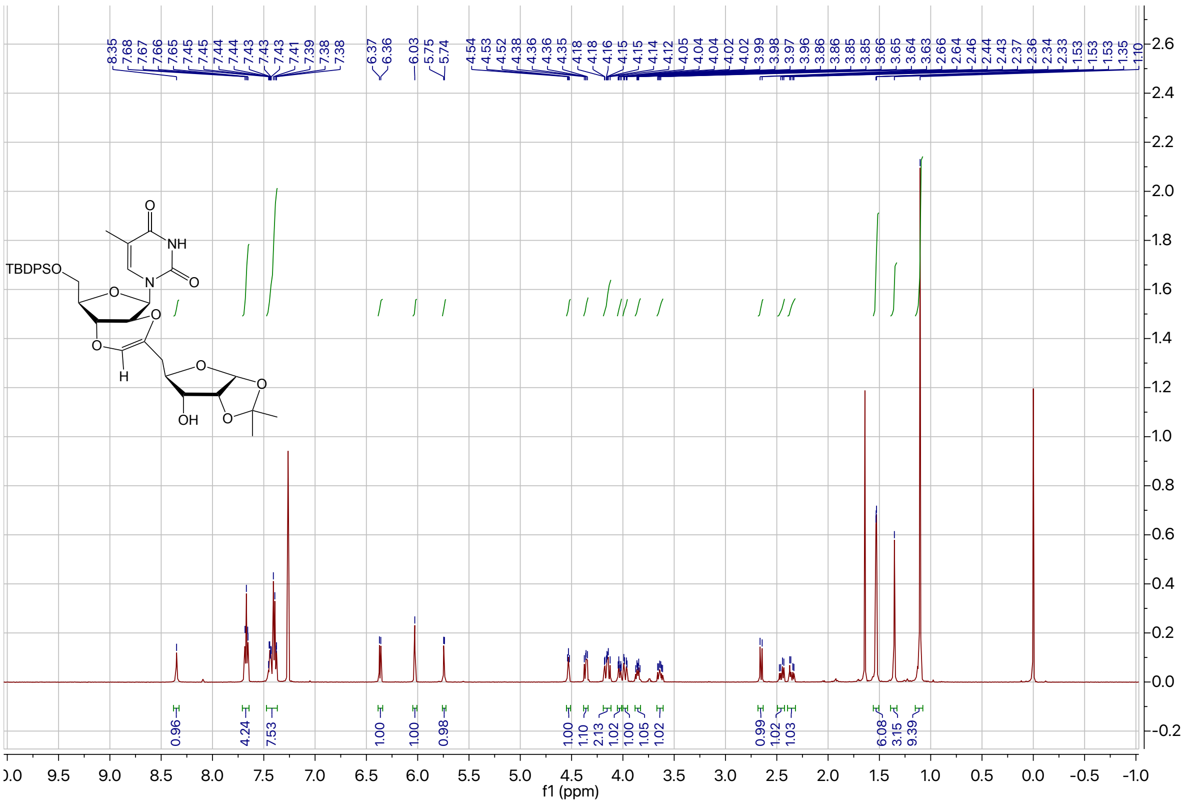

^{13}C-NMR (126 MHz, $CDCl_3$) of compound **20**

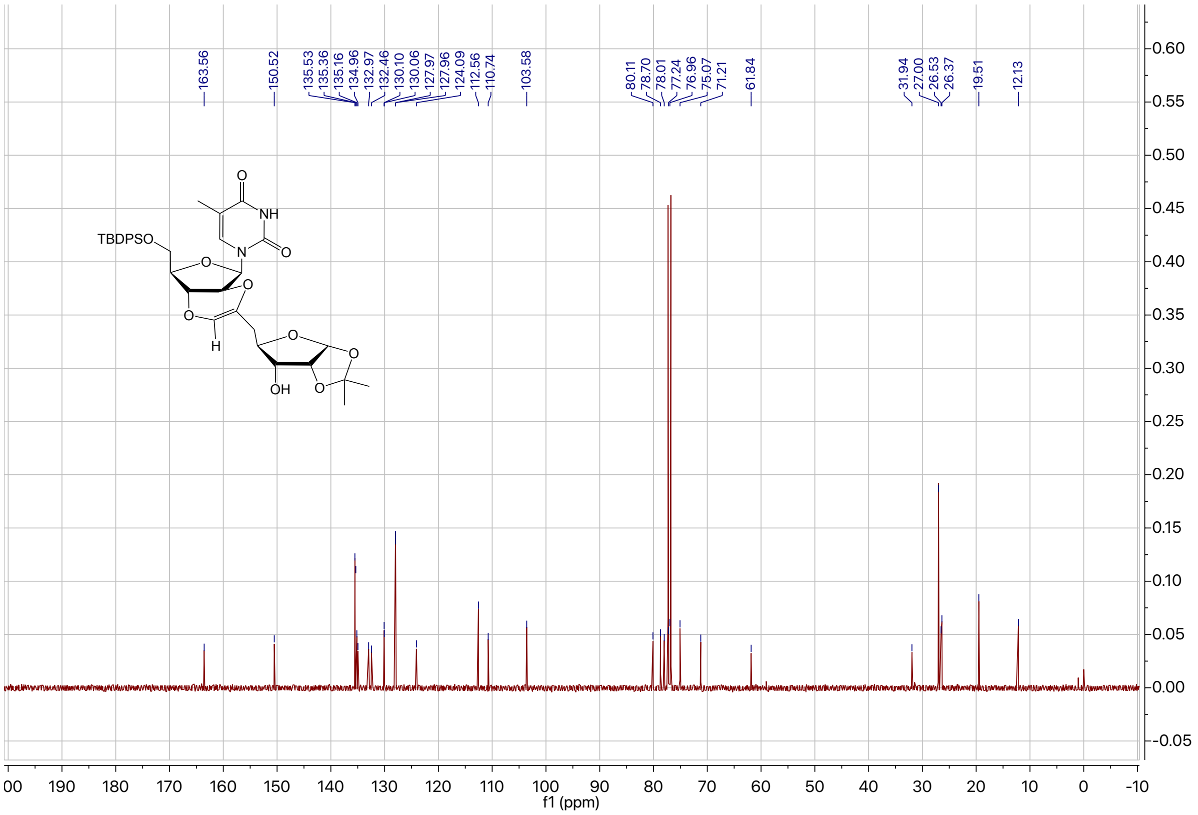

^{1}H-NMR (500 MHz, $CDCl_3$) of compound **21**

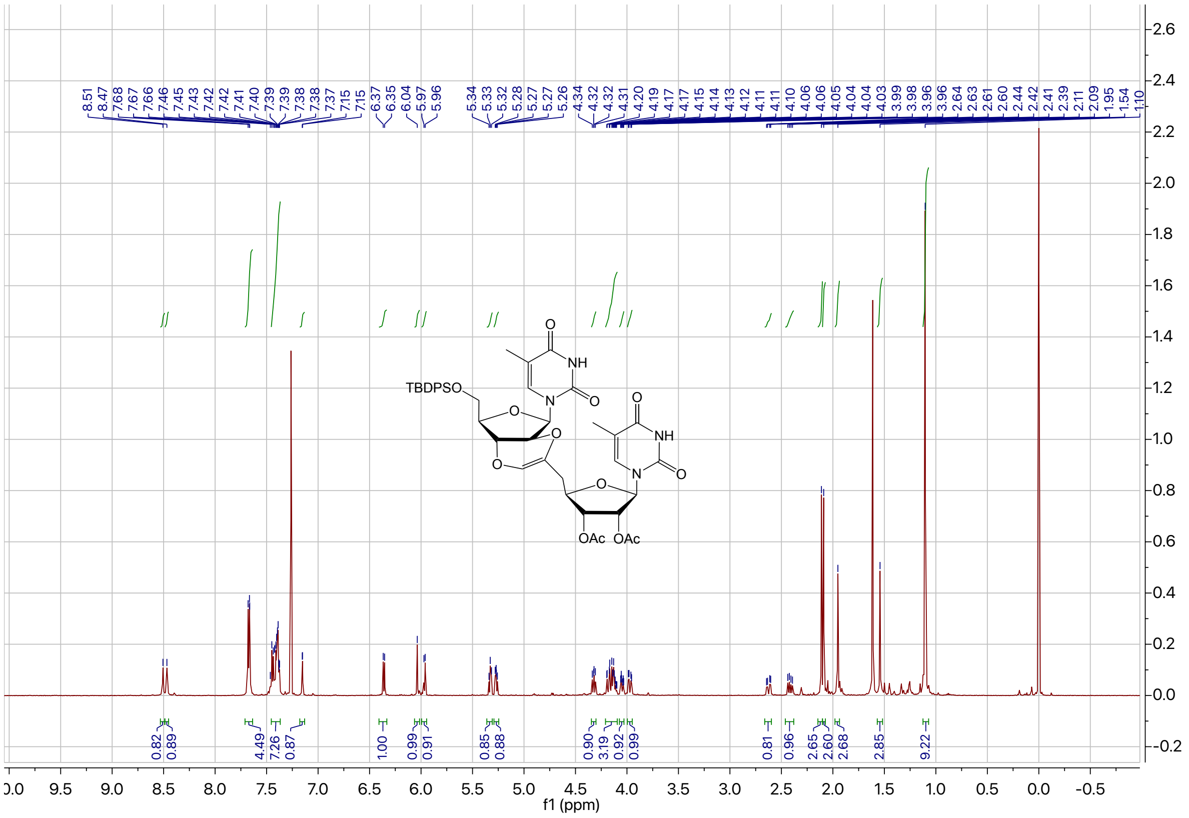

^{13}C-NMR (126 MHz, $CDCl_3$) of compound **21**

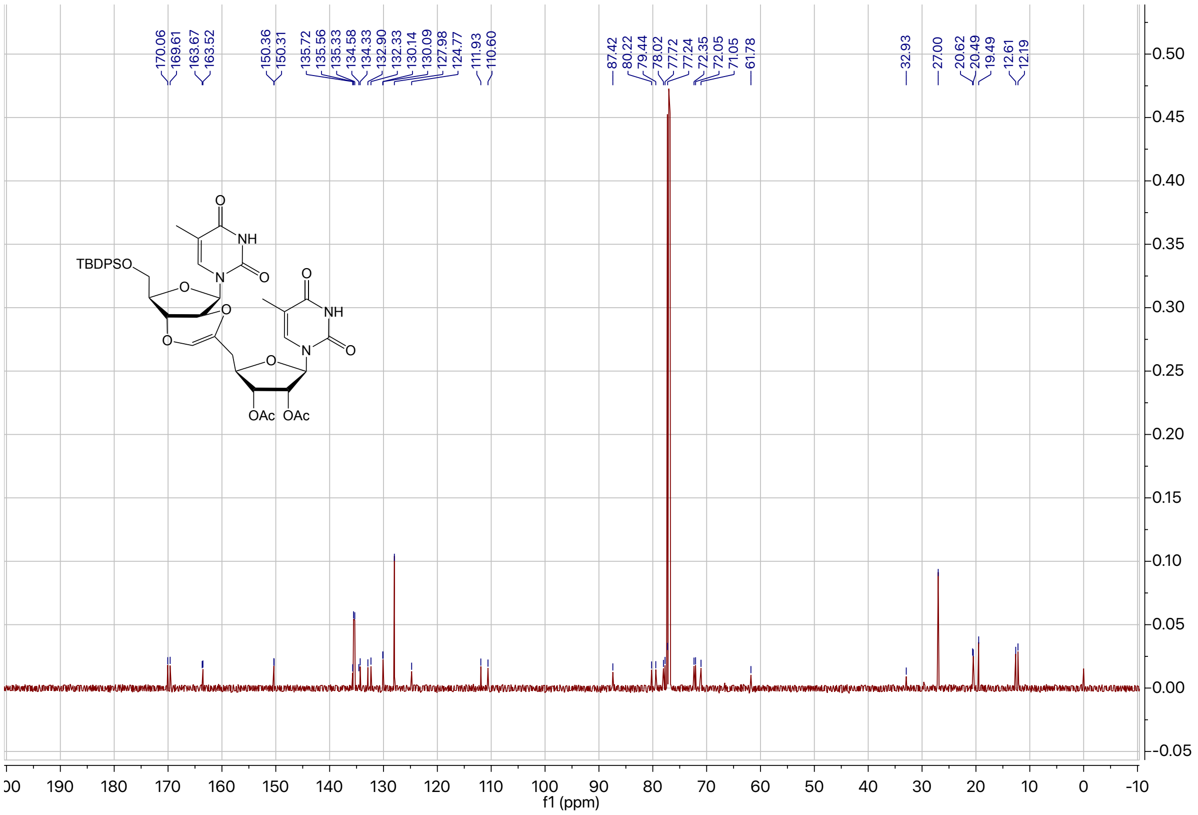

^{1}H-NMR (400 MHz, $CDCl_3$) of compound **22**

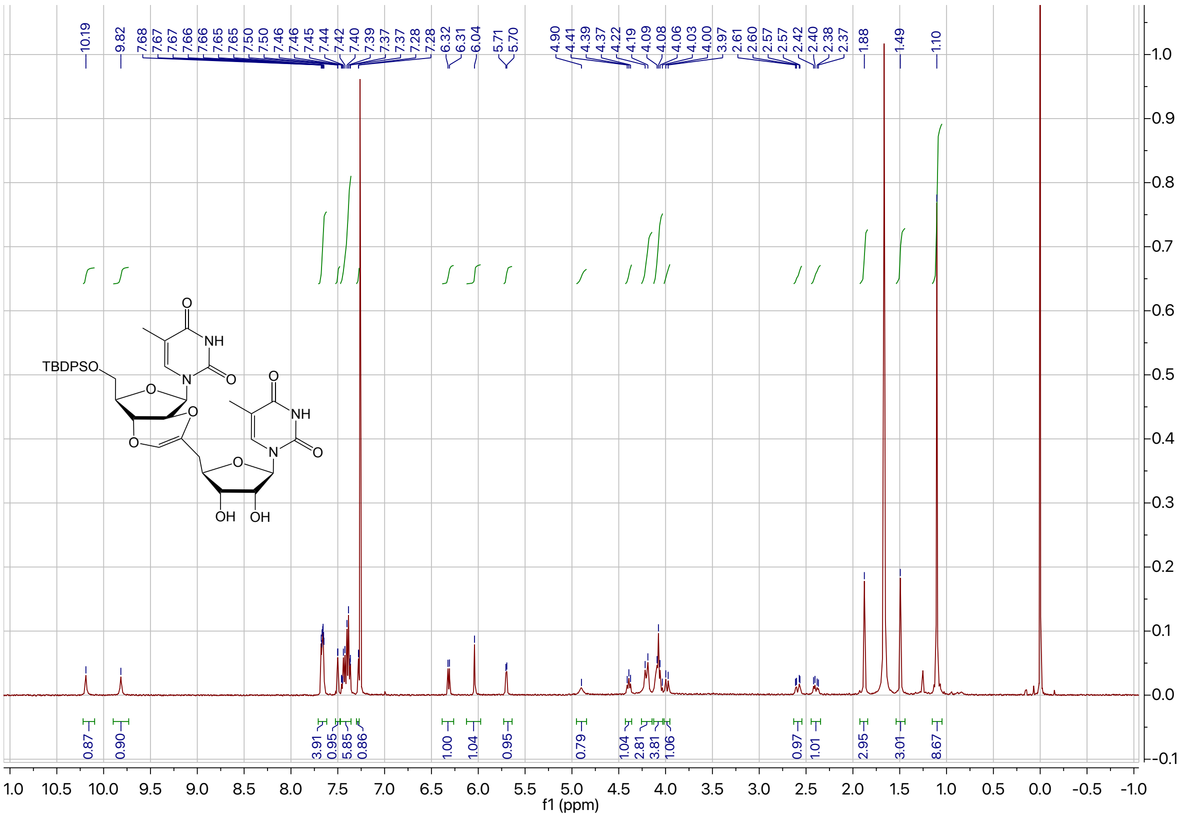

^{13}C-NMR (126 MHz, $CDCl_3$) of compound **22**

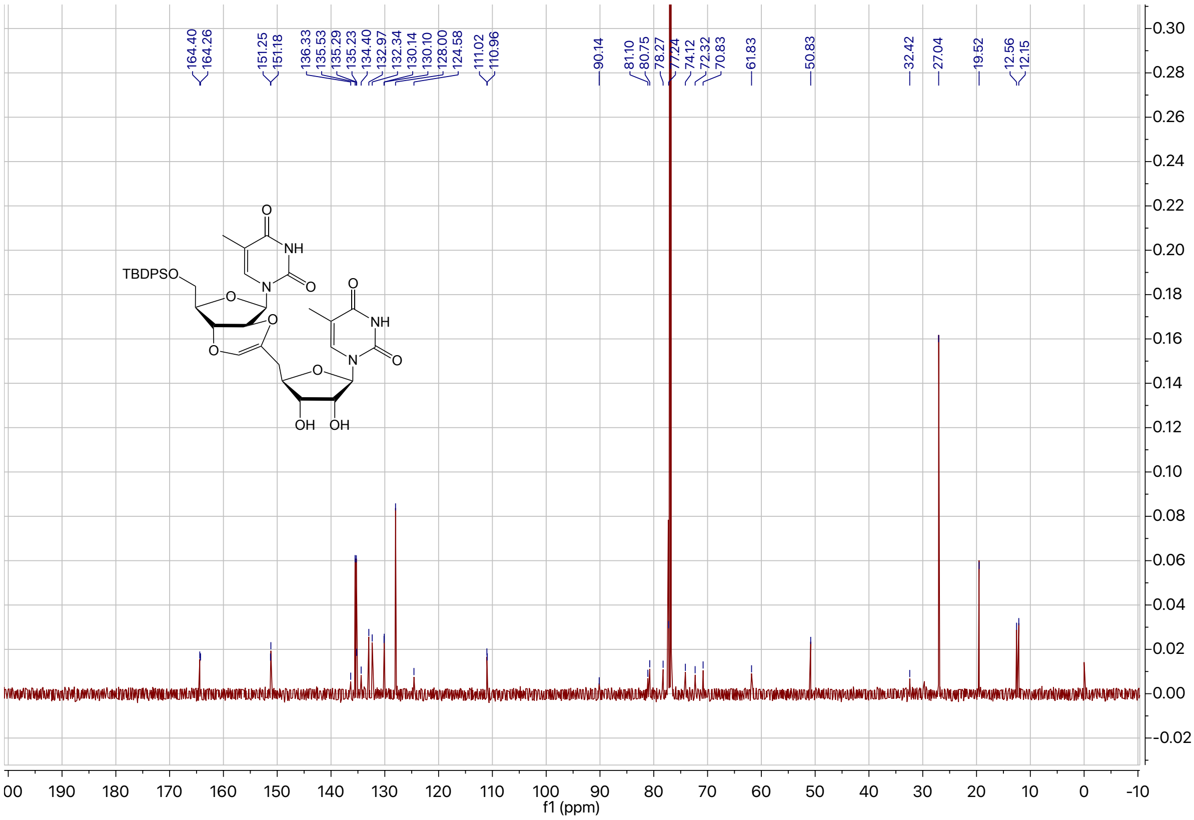

^{1}H-NMR (400 MHz, DMSO-d_6) of compound **23** (2′,3′-5,6-*trans*-BNA^{olefin})

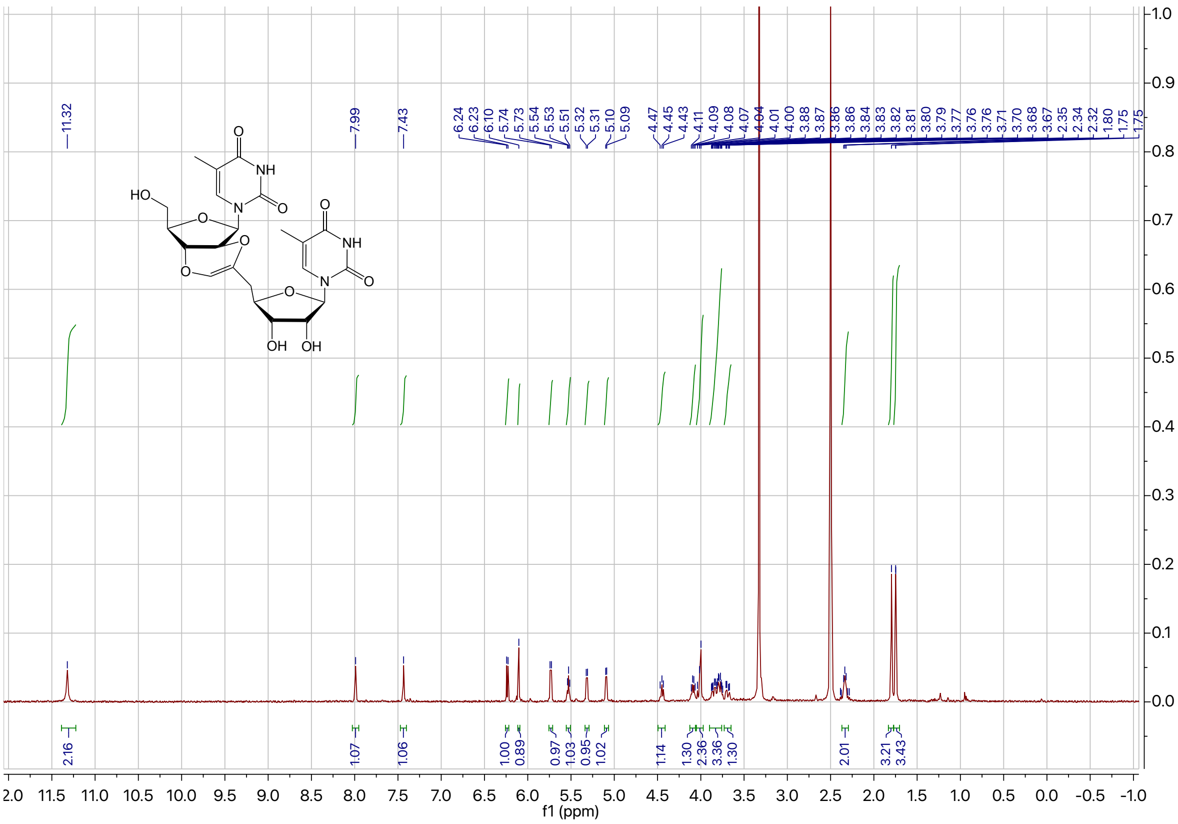

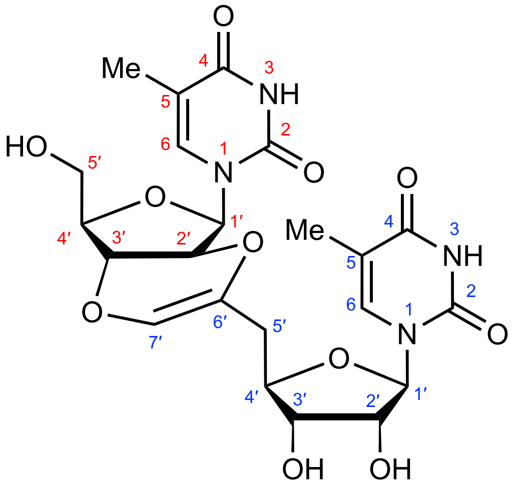

23

δ = 11.32 (s, 2H) 3-NH, 3-NH
7.99 (d, J = 1.0 Hz, 1H) 6-H
7.43 (d, J = 1.1 Hz, 1H) 6-H
6.24 (d, J = 6.5 Hz, 1H) 1′-H
6.10 (s, 1H) 7′-H
5.73 (d, J = 6.5 Hz, 1H) 1′-H
5.53 (t, J = 5.1 Hz, 1H) 5′-OH
5.32 (d, J = 6.0 Hz, 1H) 2′-OH
5.09 (d, J = 4.5 Hz, 1H) 3′-OH
4.47–4.43 (m, 1H) 4′-H
4.11–4.06 (m, 1H) 2′-H
4.04–3.99 (m, 2H) 3′-H, 2′-H,
3.88–3.75 (m, 3H) 5′-H, 3′-H, 4′-H
3.71–3.66 (m, 1H) 5′-H
2.39–2.28 (m, 2H) 5′-H
1.80 (d, J = 0.9 Hz, 3H) 5-CH_3
1.75 (d, J = 0.8 Hz, 3H) 5-CH_3

^{13}C-NMR (126 MHz, CD_3OD) of compound **23** (2′,3′-5,6-*trans*-BNA^{olefin})

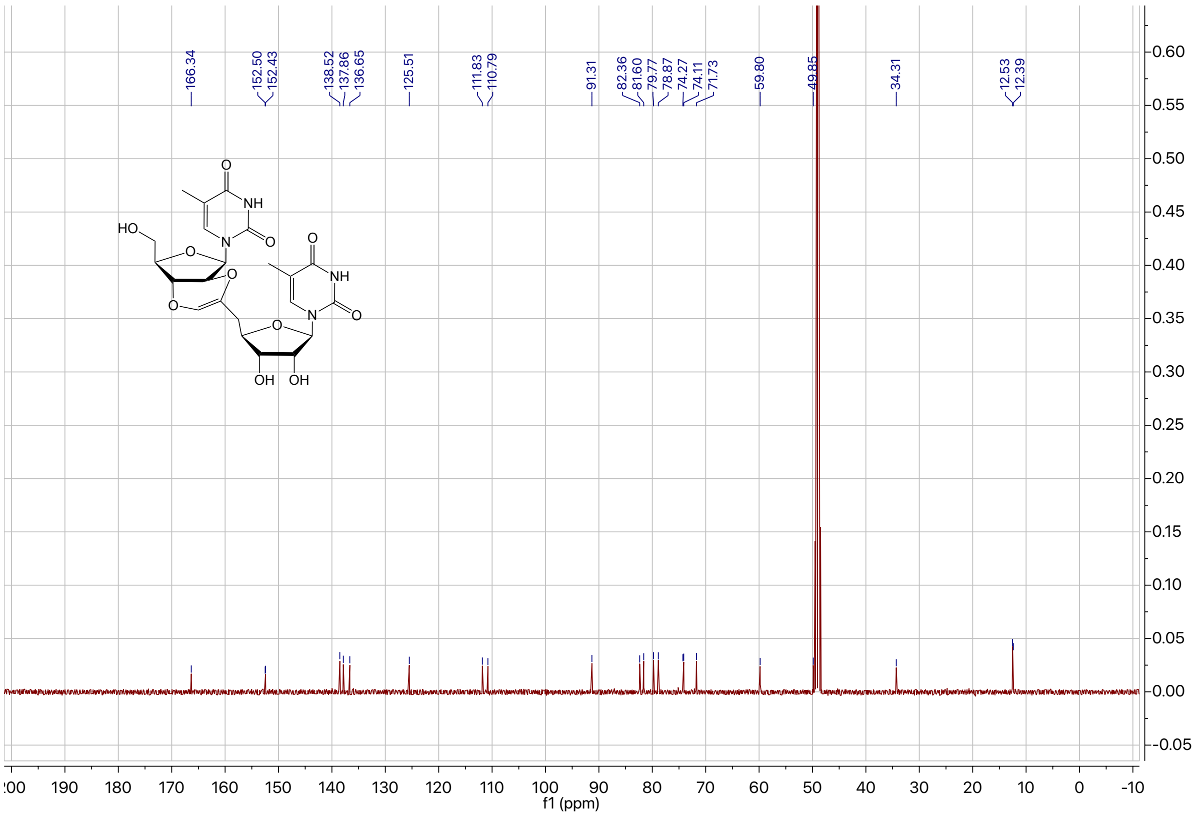

^{1}H-NMR (500 MHz, CD_3OD) of compound **24** (2′,3′-5,6-*trans*-BNA)

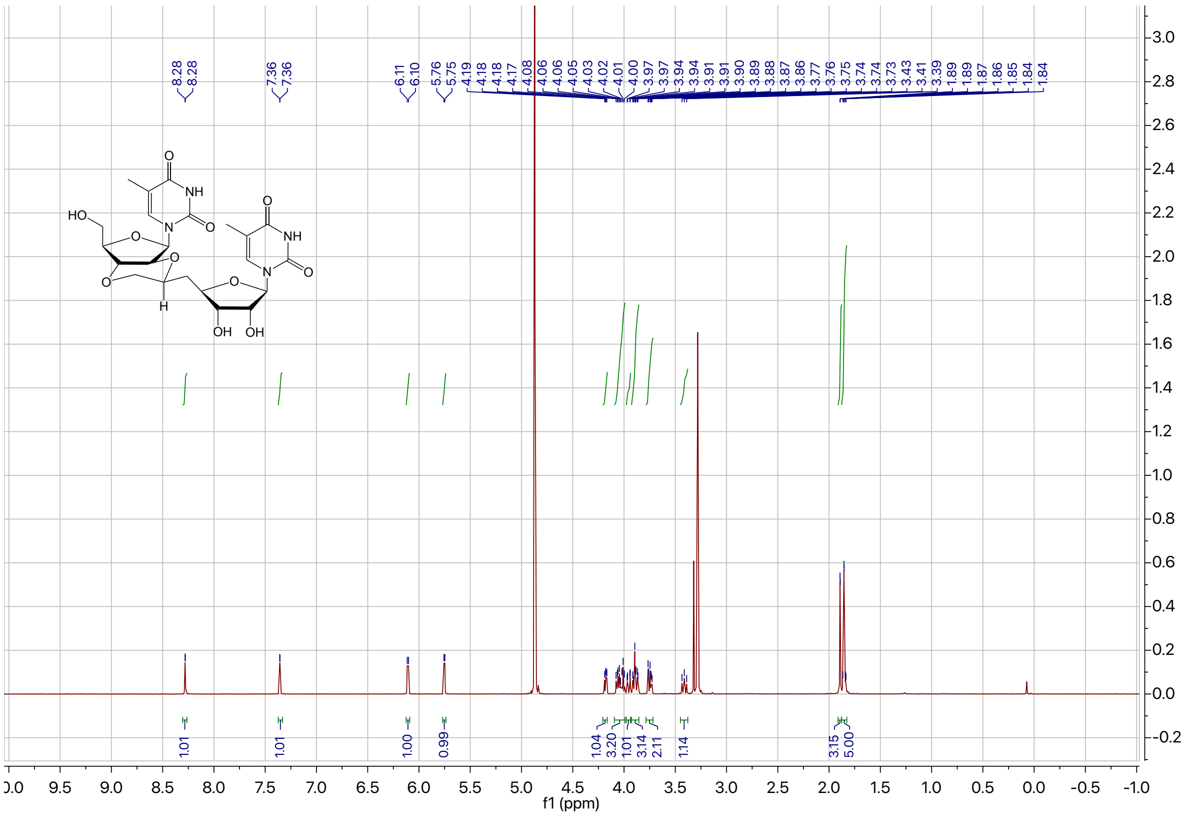

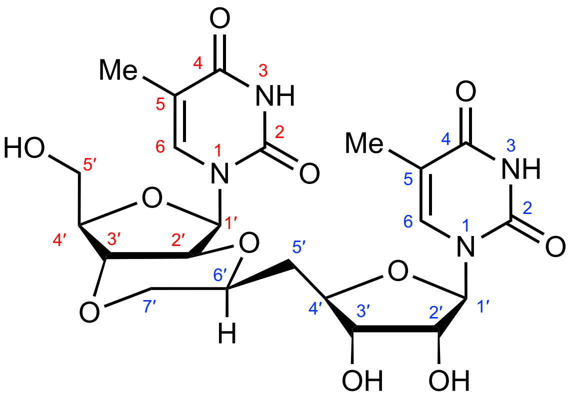

24

δ = 8.28 (d, *J* = 1.2 Hz, 1H) 6-H
7.36 (d, *J* = 1.2 Hz, 1H) 6-H
6.11 (d, *J* = 6.4 Hz, 1H) 1′-H
5.75 (d, *J* = 4.5 Hz, 1H) 1′-H
4.18 (dd, *J* = 6.0, 4.5 Hz, 1H) 2′-H
4.08–4.00 (m, 3H) 2′-H, 3′-H, 3′-H
3.95 (dd, *J* = 13.0, 2.0 Hz, 1H) 7′-H
3.93–3.86 (m, 3H) 4′-H, 5′-H, 4′-H
3.77–3.72 (m, 2H) 5′-H, 6′-H
3.41 (dd, *J* = 11.7, 11.1 Hz, 1H) 7′-H
1.89 (d, *J* = 1.2 Hz, 3H) 5-CH_3
1.83–1.87 (m, 5H) 5-CH_3, 5′-H

^{13}C-NMR (100 MHz, CD_3OD) of compound **24** (2′,3′-5,6-*trans*-BNA)

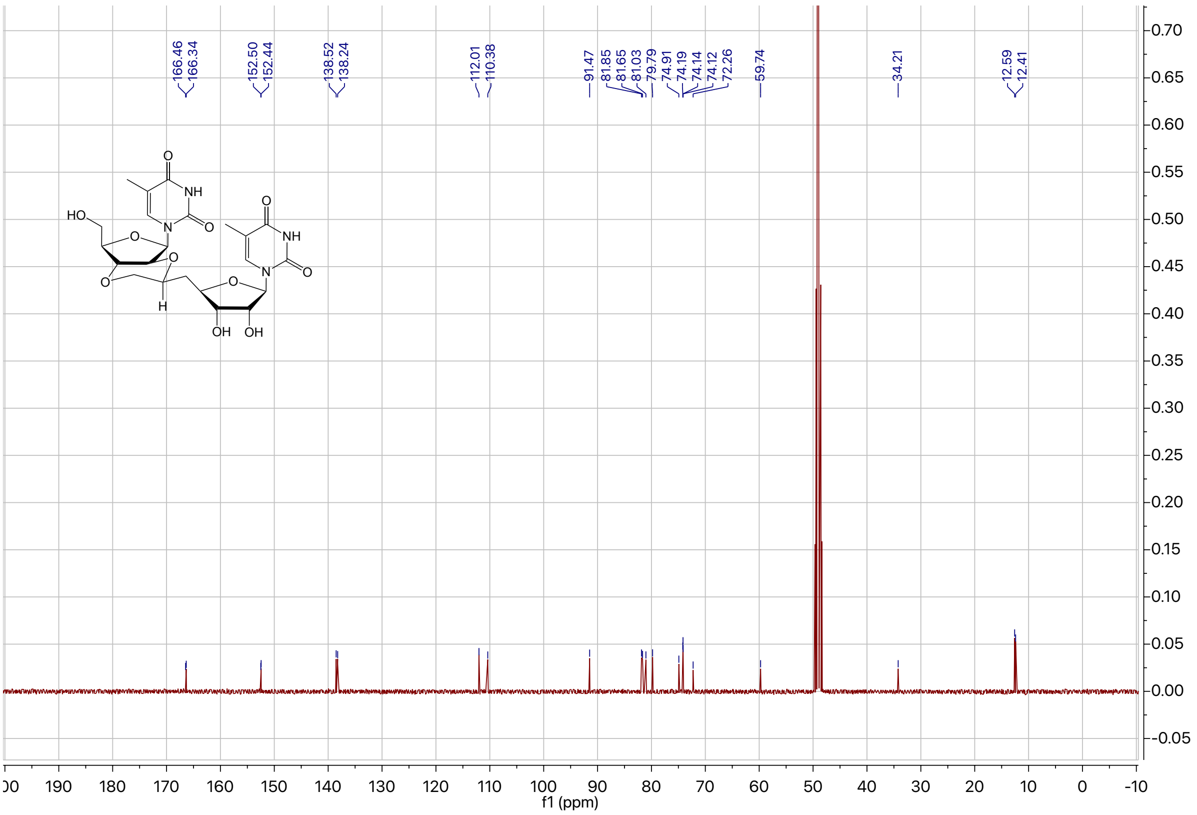

H-H COSY (500 MHz, acetone-d_6) of compound **24**

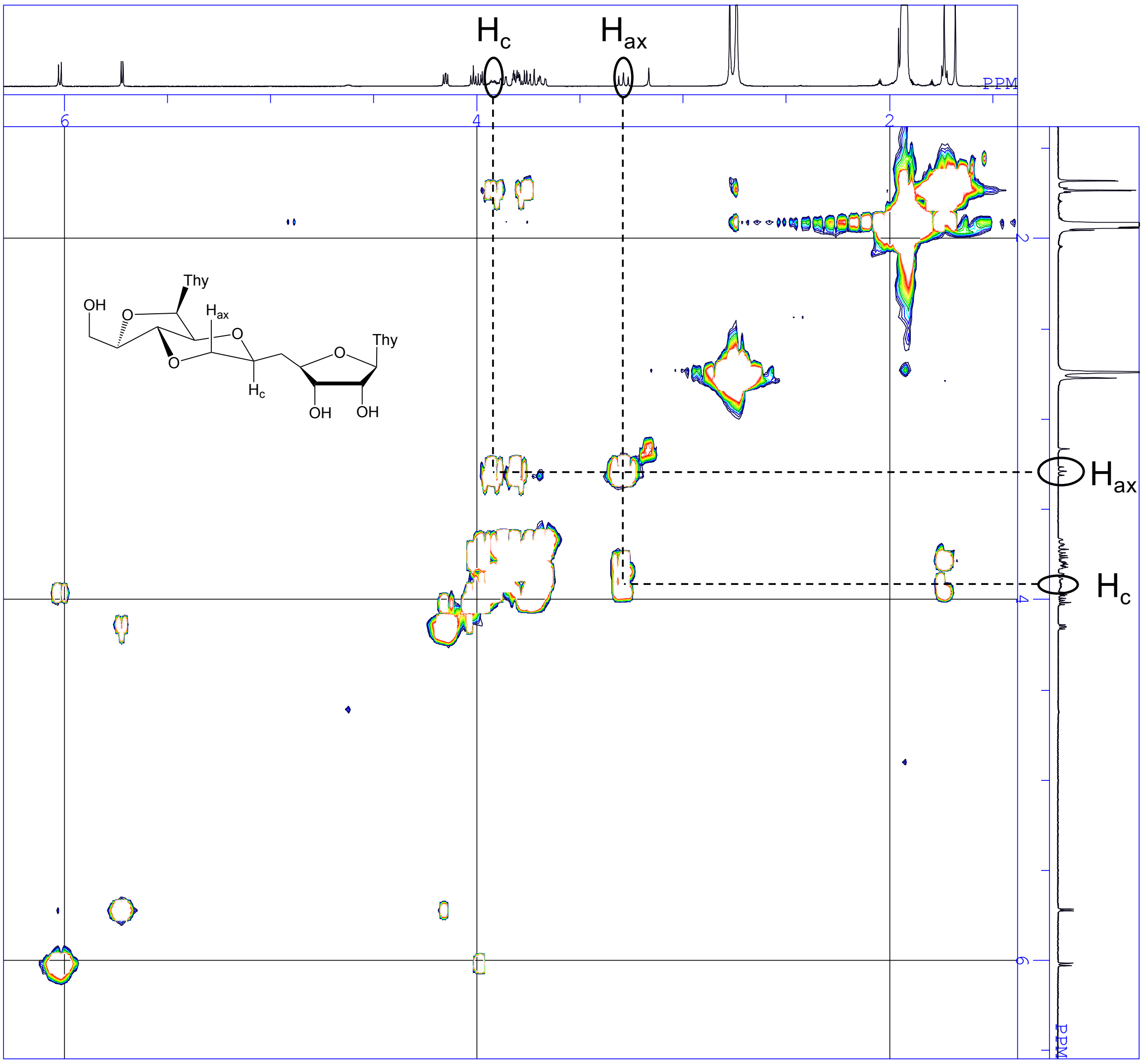

^{1}H-NMR (500 MHz, DMSO-d_6) of compound **25**

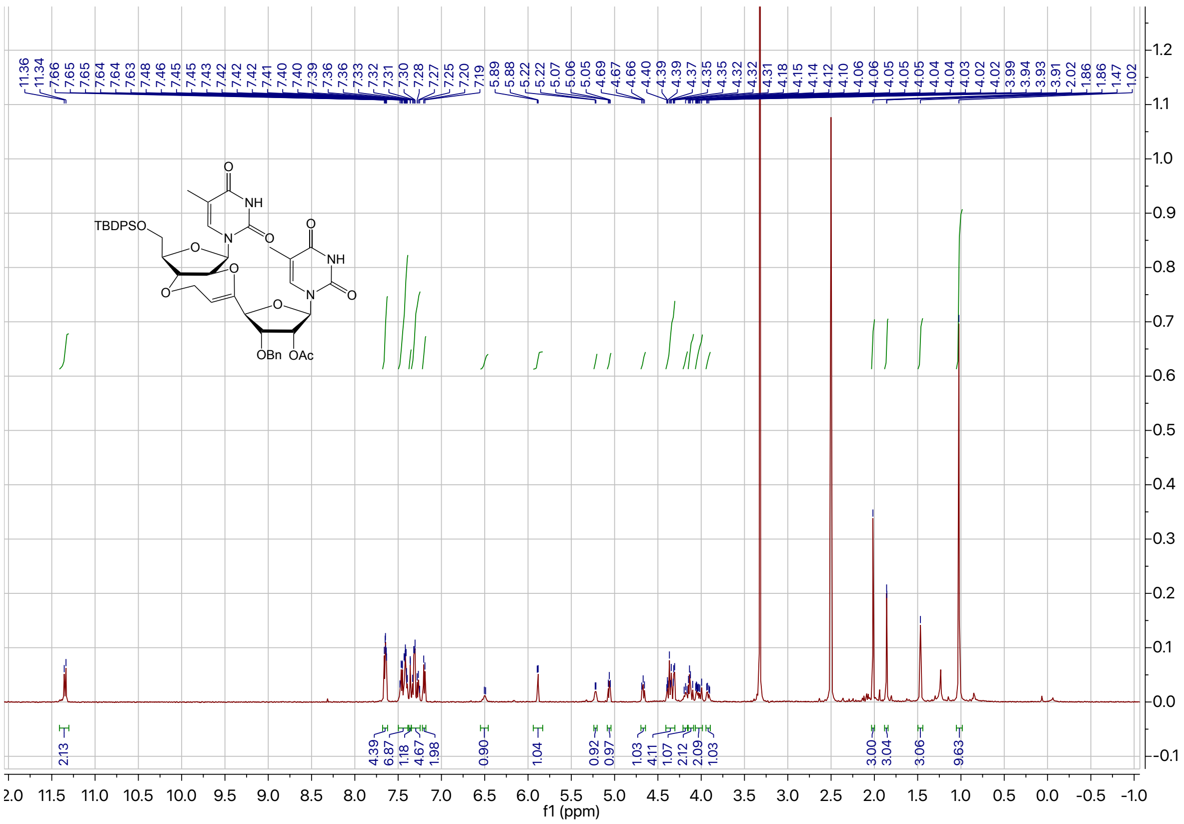

^{13}C-NMR (126 MHz, $CDCl_3$) of compound **25**

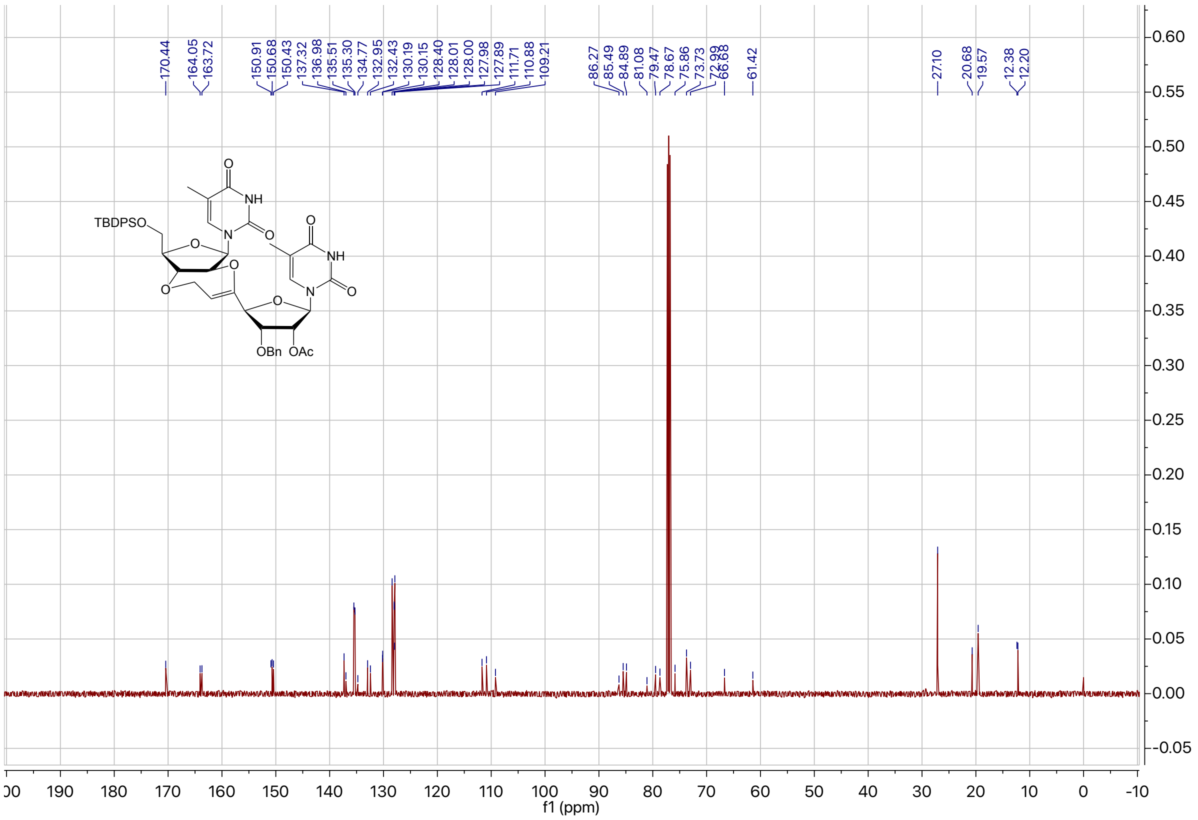

^{1}H-NMR (500 MHz, $CDCl_3$) of compound **26**

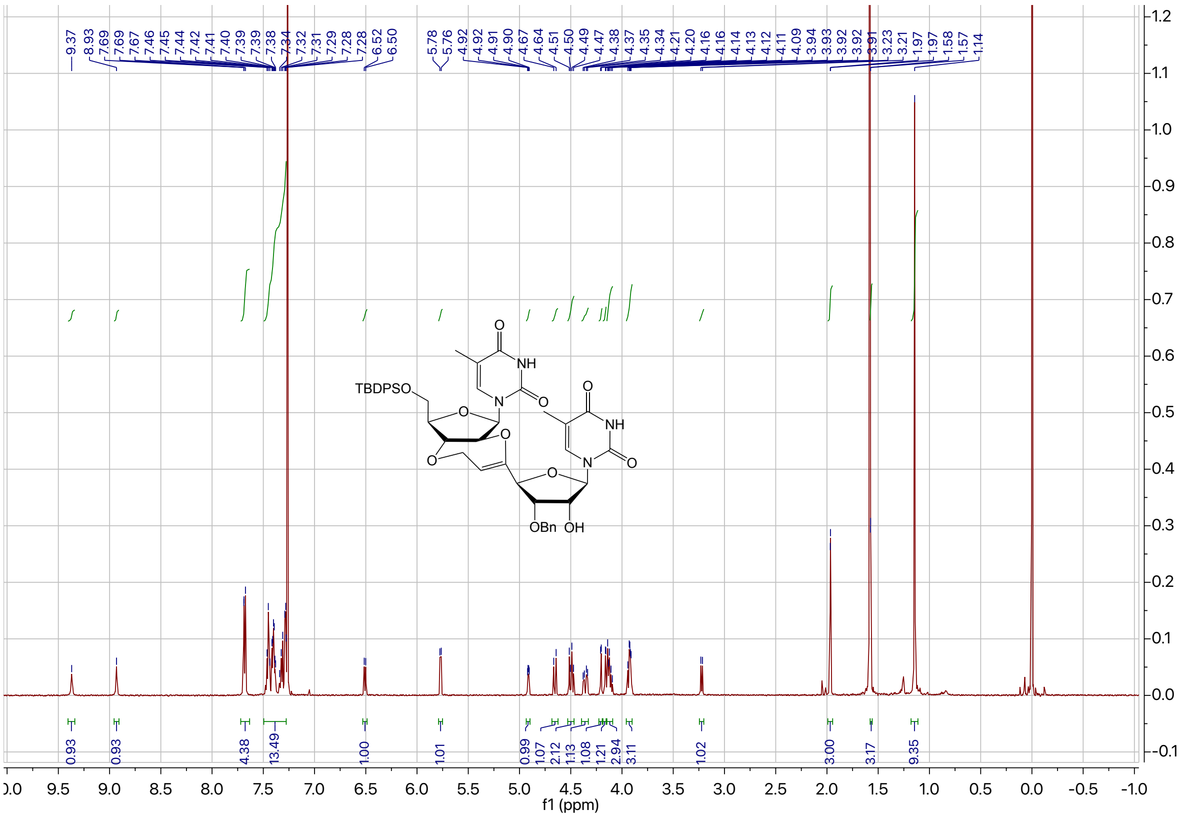

^{13}C-NMR (126 MHz, $CDCl_3$) of compound **26**

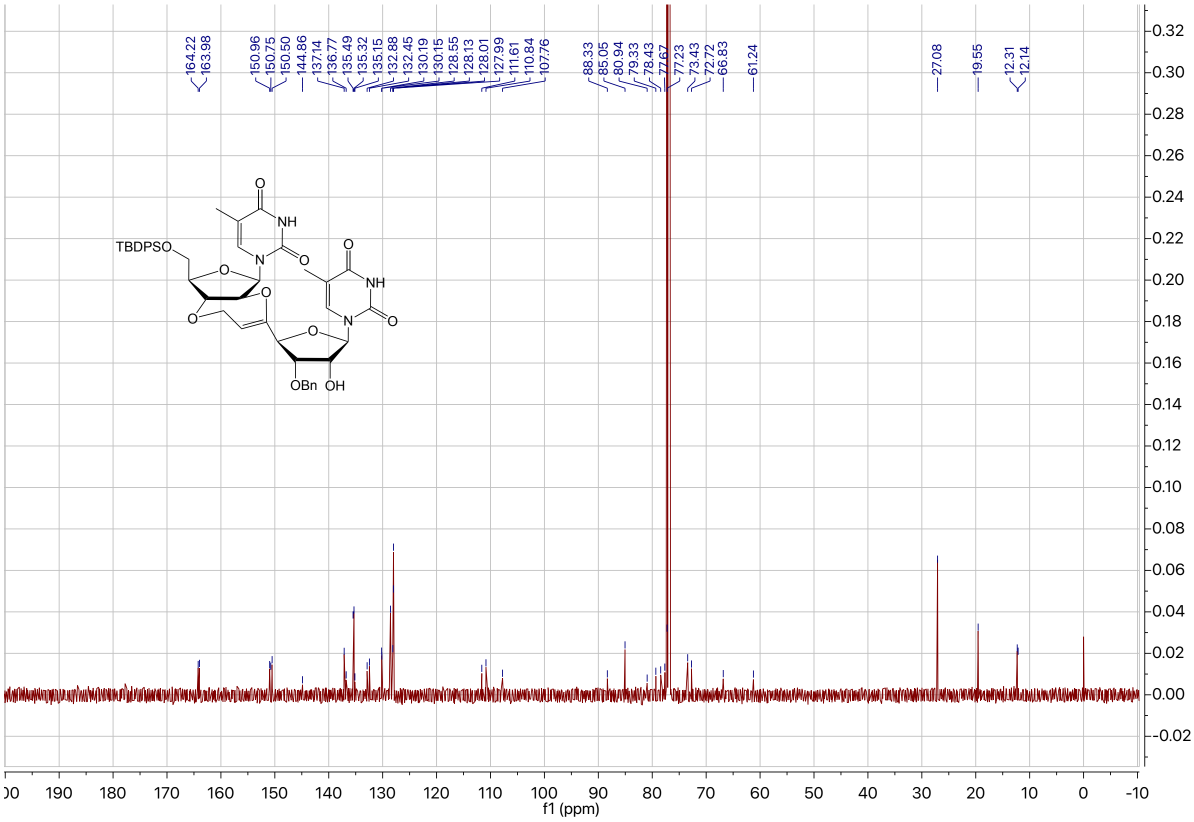

^{1}H-NMR (500 MHz, CD_3OD) of compound **27**

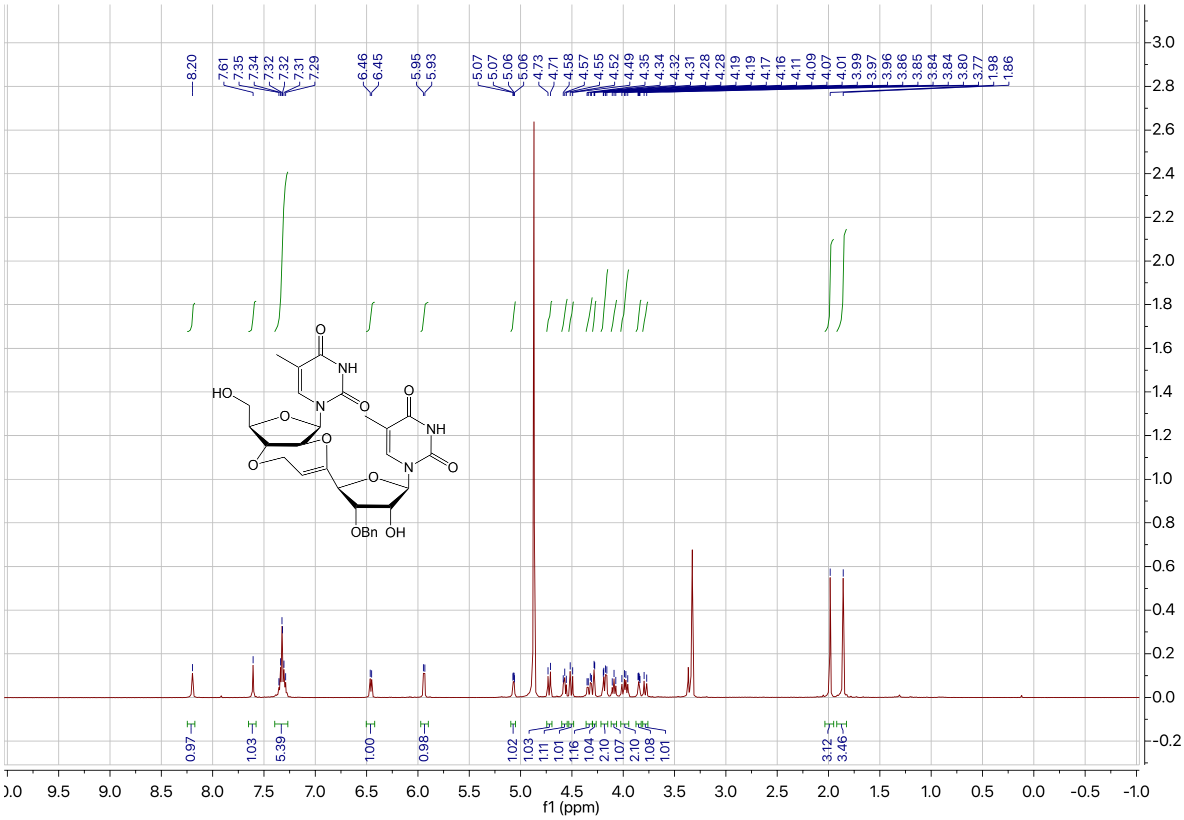

^{13}C-NMR (126 MHz, CD_3OD) of compound **27**

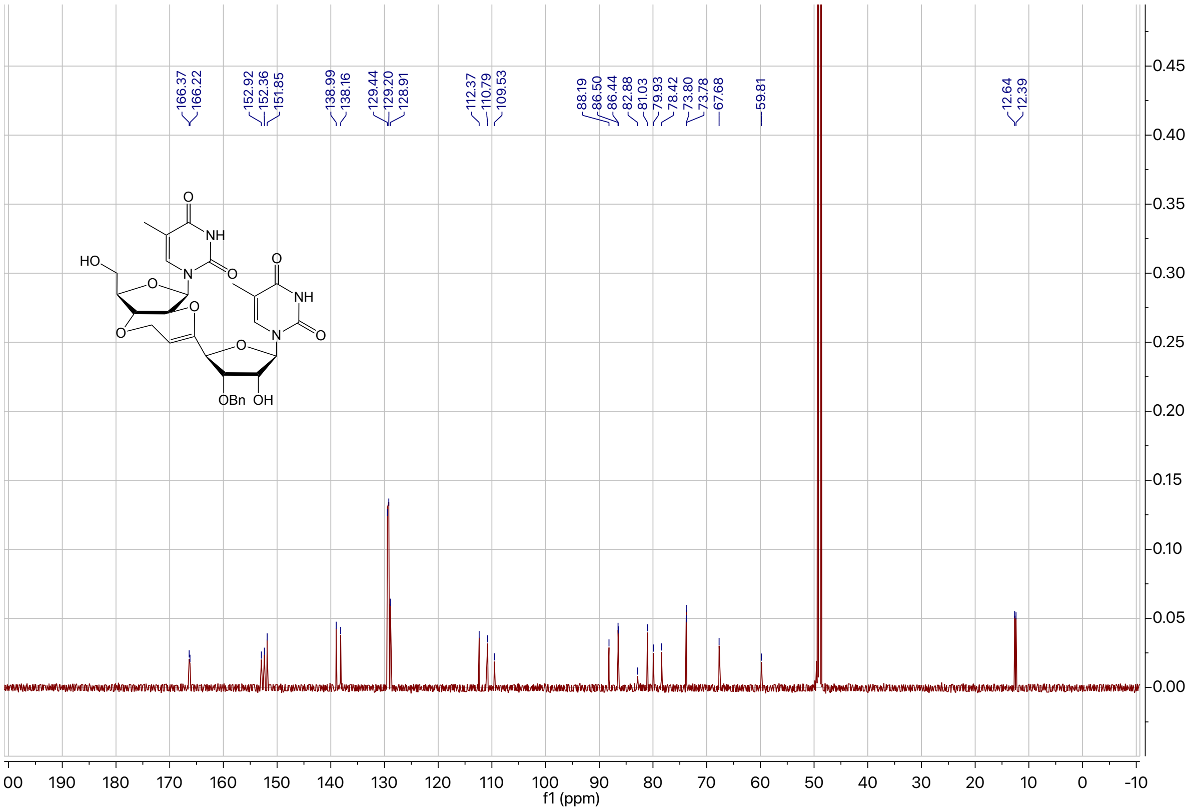

^{1}H-NMR (400 MHz, CD_3OD) of compound **28** (*R*-isomer of 2′,3′-5,7-*trans*-BNA)

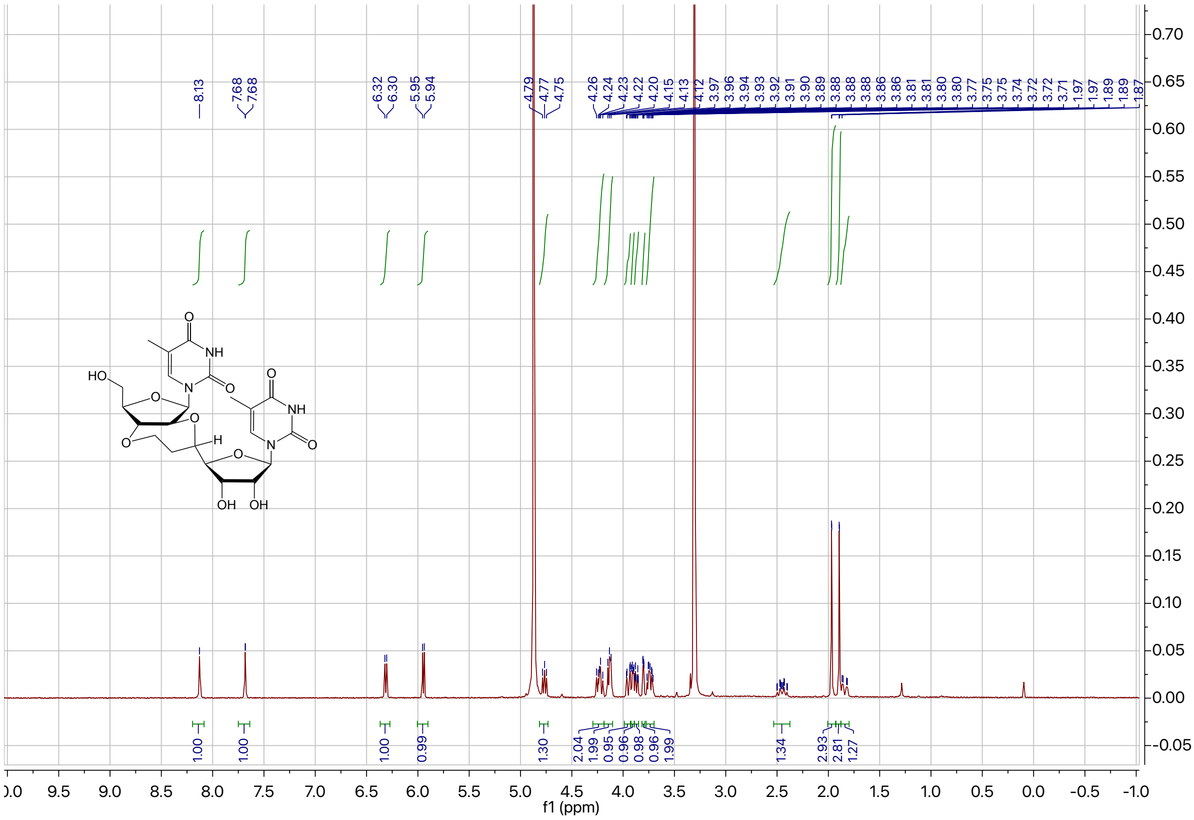

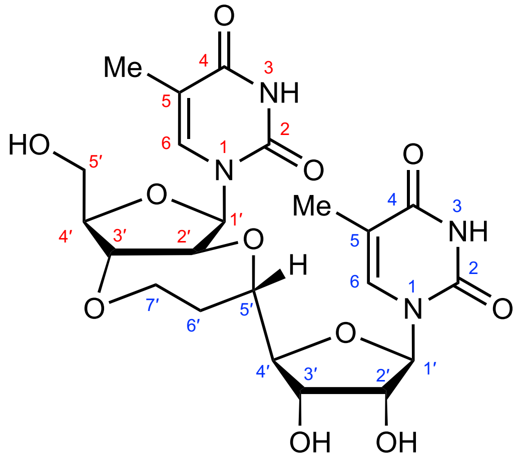

28

δ = 8.13 (d, *J* = 1.0 Hz, 1H) 6-H
7.68 (d, *J* = 1.2 Hz, 1H) 6-H
6.31 (d, *J* = 7.1 Hz, 1H) 1′-H
5.95 (d, *J* = 6.7 Hz, 1H) 1′-H
4.77 (dd, *J* = 8.0, 7.5 Hz, 1H) 2′-H
4.27–4.18 (m, 2H) 2′-H, 7′-H
4.16–4.10 (m, 2H) 3′-H, 5′-H
3.95 (dd, *J* = 12.8, 2.0 Hz, 1H) 5′-H
3.91 (dd, *J* = 6.2, 3.5 Hz, 1H) 3′-H
3.87 (dt, *J* = 9.1, 2.0 Hz, 1H) 7′-H
3.80 (dd, *J* = 3.5, 1.0 Hz, 1H) 4′-H
3.77–3.70 (m, 2H) 4′-H, 5′-H
2.51–2.39 (m, 1H) 6′-H
1.97 (d, *J* = 1.1 Hz, 3H) 5-CH_3
1.89 (d, *J* = 1.1 Hz, 3H) 5-CH_3
1.87–1.80 (m, 1H) 6′-H

^{13}C-NMR (100 MHz, CD_3OD) of compound **28** (*R*-isomer of 2′,3′-5,7-*trans*-BNA)

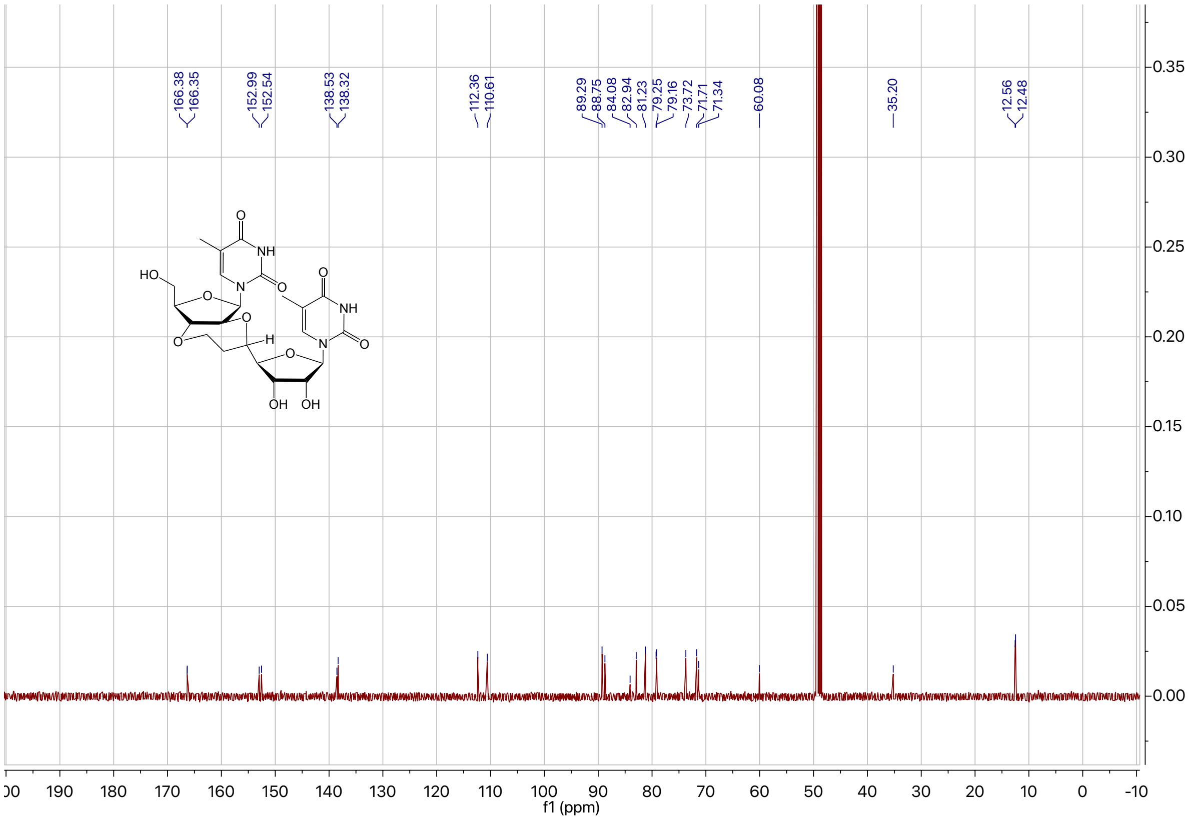

H-H COSY (400 MHz, acetone-d_6) of compound **28** (*R*-isomer of 2′,3′-5,7-*trans*-BNA)

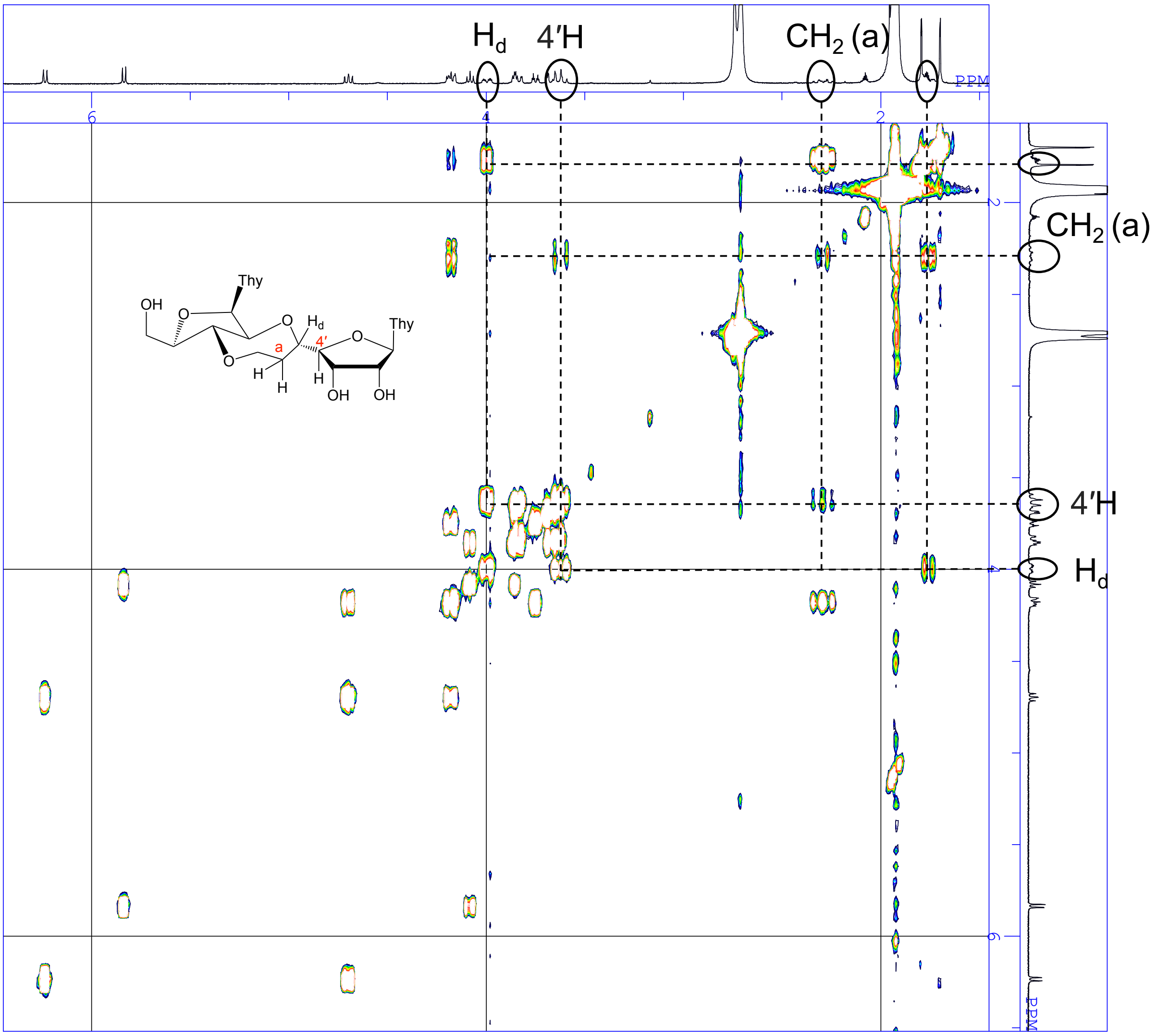

NOESY (500 MHz, acetone-d_6) of compound **28** (*R*-isomer of 2′,3′-5,7-*trans*-BNA)

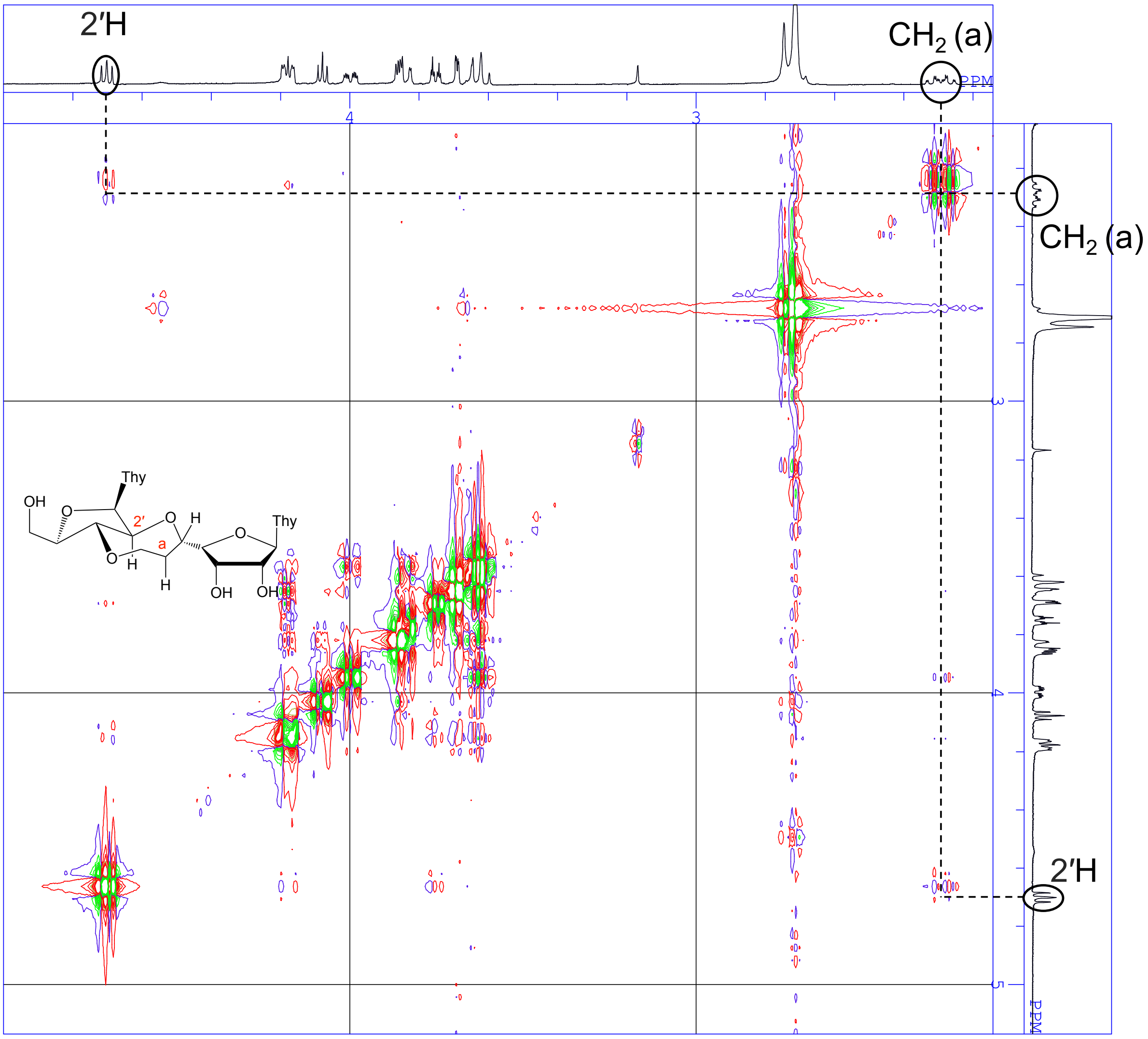

^{1}H-NMR (400 MHz, CD_3OD) of compound **29** (*S*-isomer of 2′,3′-5,7-*trans*-BNA)

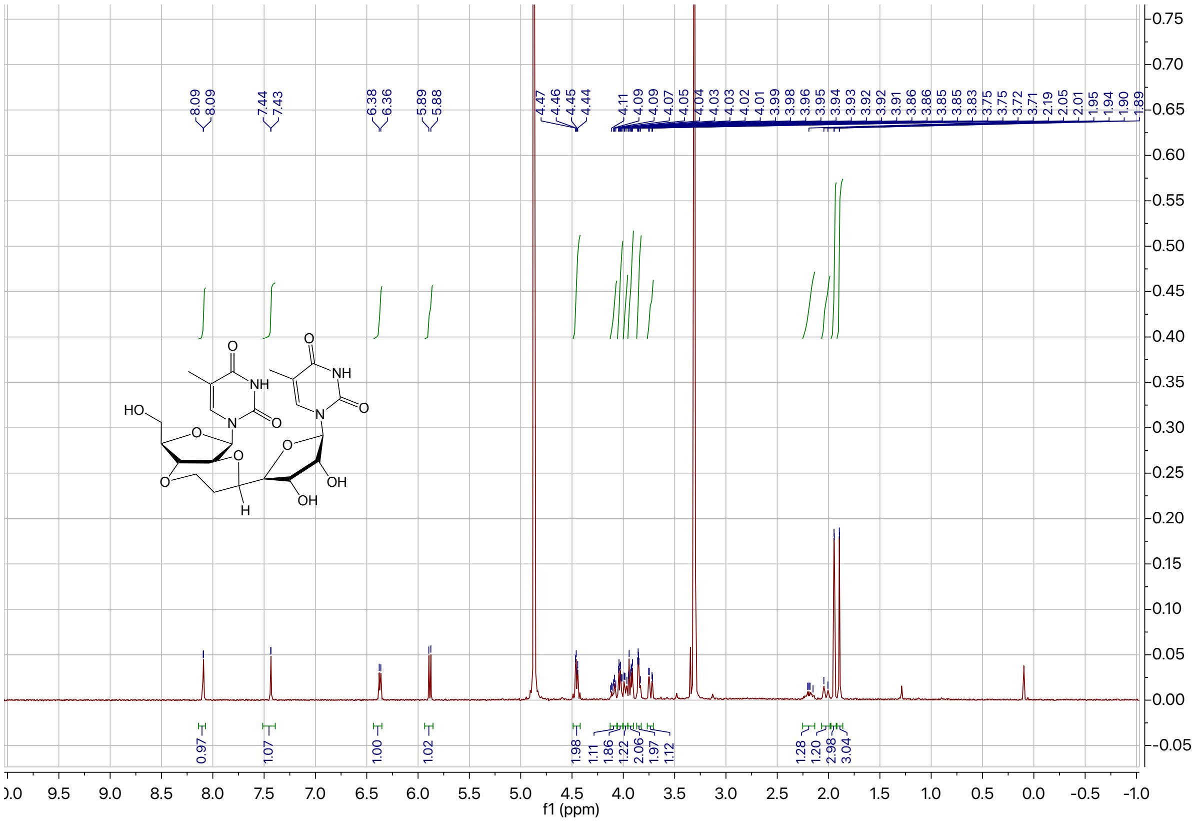

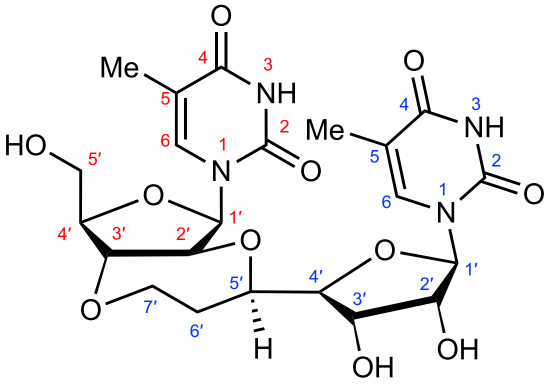

29

δ = 8.09 (d, *J* = 1.2 Hz, 1H) 6-H
7.43 (d, *J* = 1.2 Hz, 1H) 6-H
6.37 (d, *J* = 6.4 Hz, 1H) 1′-H
5.89 (d, *J* = 7.9 Hz, 1H) 1′-H
4.49–4.42 (m, 2H) 2′-H, 2′-H
4.13–4.07 (m, 1H) 7′-H
4.05–4.01 (m, 2H) 3′-H, 5′-H
4.00–3.96 (m, 1H) 5′-H
3.95–3.90 (m, 2H) 3′-H, 7′-H
3.87–3.82 (m, 2H) 4′-H, 4′-H
3.73 (dd, *J* = 12.9, 2.3 Hz, 1H) 5′-H
2.24–2.13 (m, 1H) 6′-H
2.02 (ddd, *J* = 16.2, 5.0, 3.6 Hz, 1H) 6′-H
1.95 (d, *J* = 1.1 Hz, 3H) 5-CH_3
1.89 (d, *J* = 1.1 Hz, 3H) 5-CH_3

^{13}C-NMR (100 MHz, CD_3OD) of compound **29** (*S*-isomer of 2′,3′-5,7-*trans*-BNA)

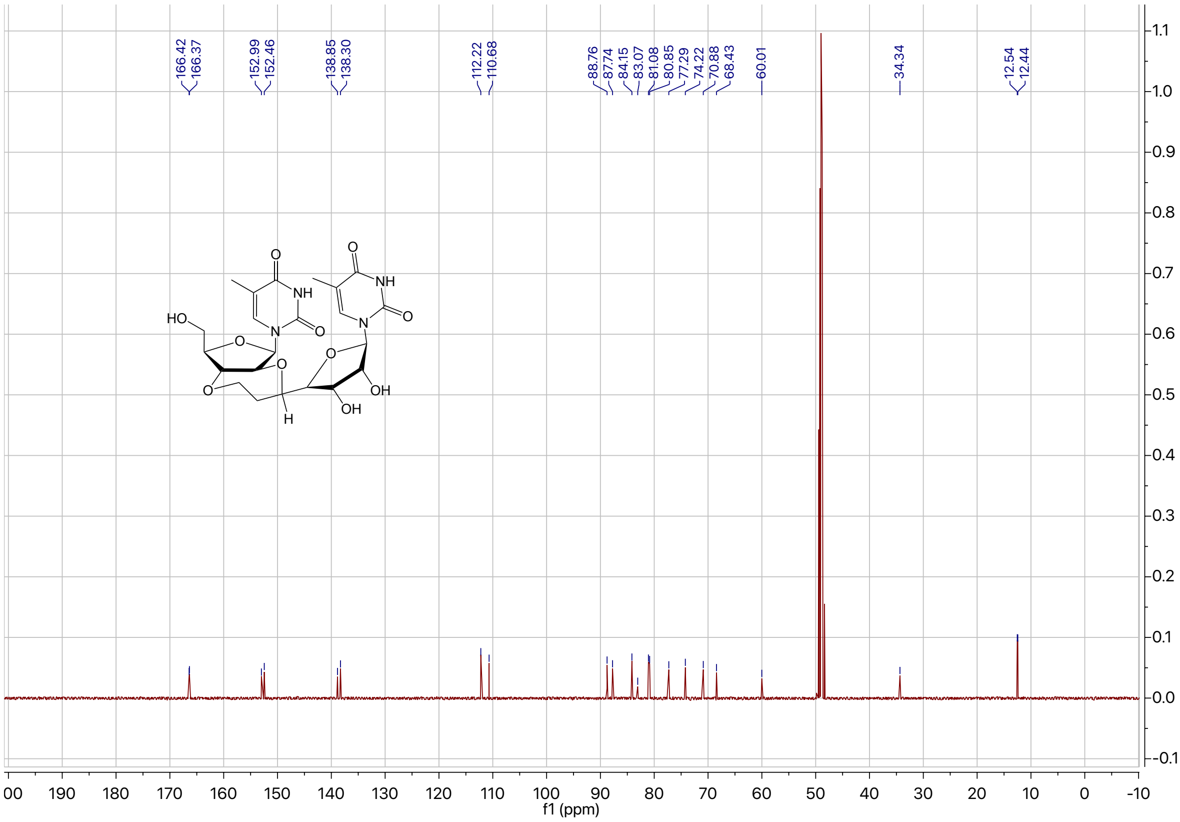

H-H COSY (400 MHz, CD_3CN) of compound **29** (*S*-isomer of 2′,3′-5,7-*trans*-BNA)

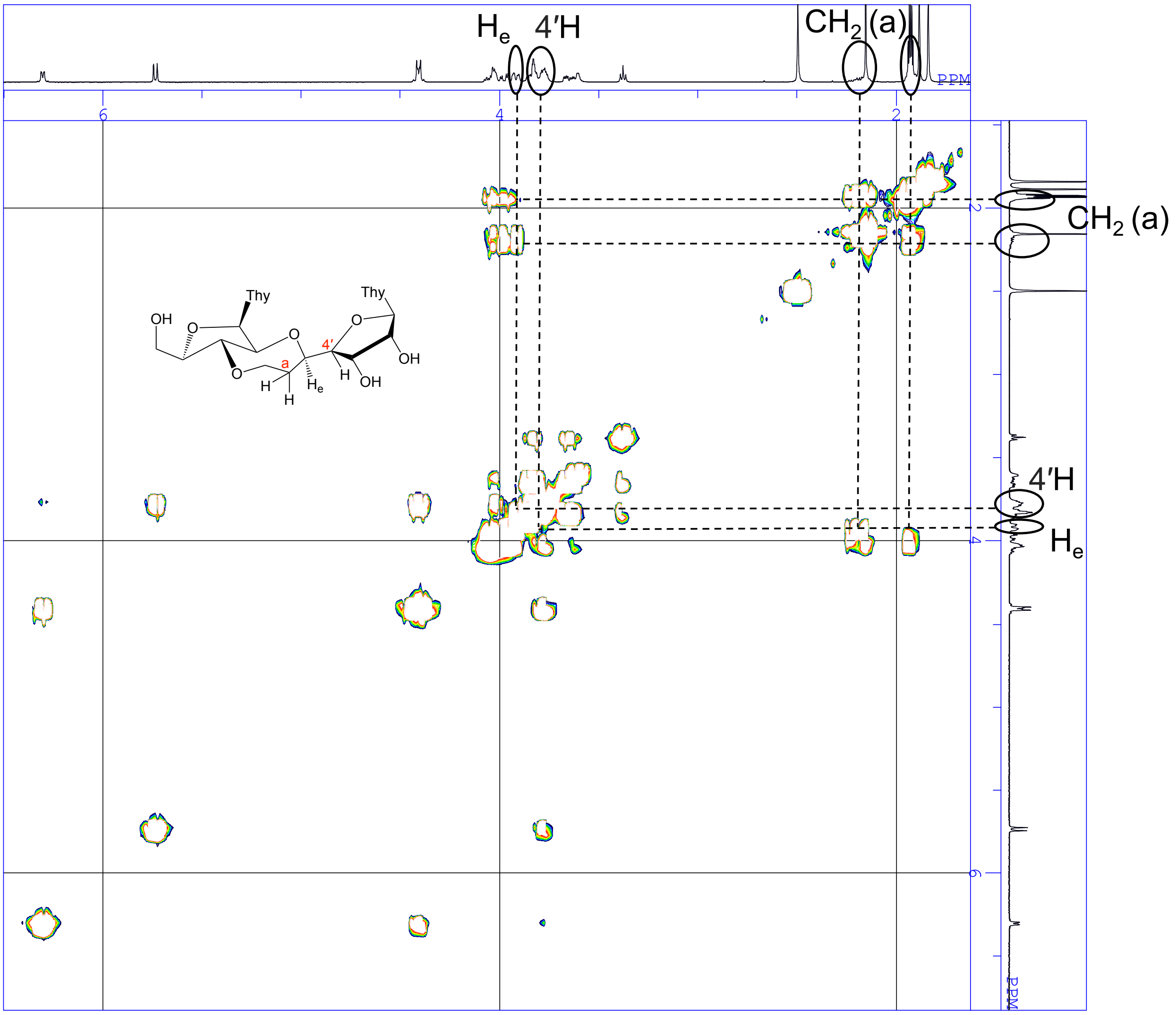

NOESY (400 MHz, CD_3CN) of compound **29** (*S*-isomer of 2′,3′-5,7-*trans*-BNA)

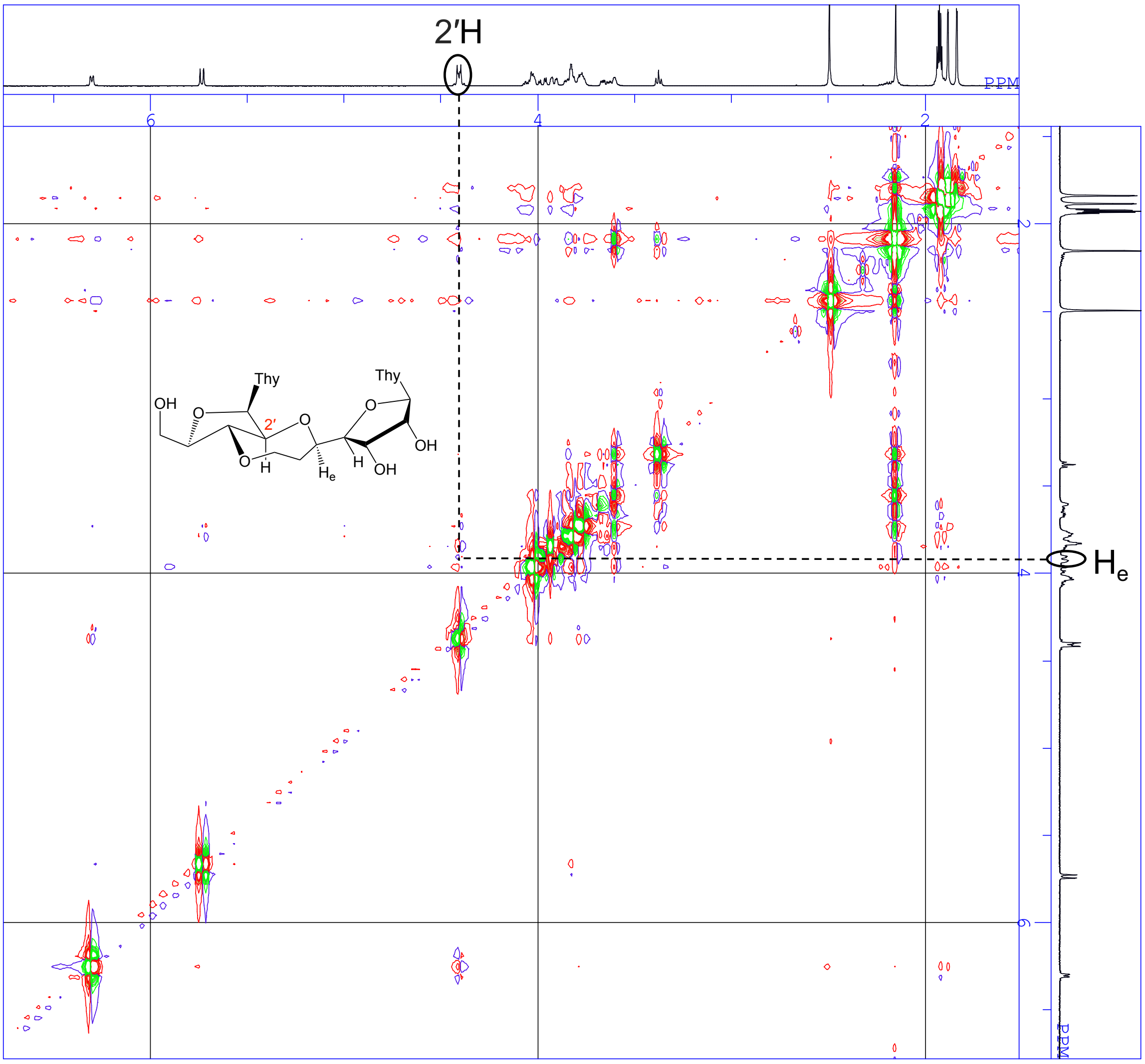

2. *Ab initio* calculation data (compounds 23a, 24a, 28a, and 29a)

1) Compound **23a**

Energy: −1065.818290 hartrees

Atomic charges

Atom Label	Natural charge	Mulliken charge	Electrostatic charge
**C1**	+0.037	−0.18	−0.259
**C2**	+0.316	−0.121	−0.089
**C3**	+0.08	−0.445	+0.032
**C4**	+0.07	−0.271	+0.343
**C5**	+0.093	−0.041	−0.029
**C6**	+0.235	+0.296	+0.306
**C7**	−0.61	−0.51	−0.857
**C8**	−0.046	−0.404	−0.051
**C9**	+0.063	+0.032	−0.323
**C10**	−0.172	+0.047	+0.136
**C11**	+0.674	+0.073	+0.604
**C12**	+0.847	+0.46	+0.613
**C13**	−0.594	−0.749	−0.79
**H1**	+0.22	+0.181	+0.26
**H2**	+0.207	+0.156	+0.23
**H3**	+0.225	+0.19	+0.233
**H4**	+0.234	+0.234	+0.19
**H5**	+0.217	+0.164	+0.239
**H6**	+0.202	+0.233	+0.143
**H7**	+0.207	+0.232	+0.11
**H8**	+0.221	+0.179	+0.256
**H9**	+0.176	+0.177	+0.109
**H10**	+0.197	+0.196	+0.112
**H11**	+0.471	+0.261	+0.425
**H12**	+0.198	+0.194	+0.149
**H13**	+0.21	+0.157	+0.22
**H14**	+0.228	+0.149	+0.253
**H15**	+0.225	+0.189	+0.234
**H17**	+0.43	+0.395	+0.392
**N1**	−0.508	+0.04	+0.05
**N2**	−0.651	−0.463	−0.618
**O1**	−0.607	−0.213	−0.33
**O2**	−0.555	+0.105	−0.262
**O3**	−0.552	−0.045	−0.363
**O4**	−0.736	−0.234	−0.59
**O5**	−0.61	−0.335	−0.527
**O6**	−0.642	−0.329	−0.55

Calculated bond orders

Bond	Löwdin	Mulliken	Bond	Löwdin	Mulliken
**C1 - C6**	1.752	1.823	**C9 - C10**	1.675	1.807
**C1 - H2**	0.856	0.965	**C9 - H14**	0.803	0.912
**C1 - O3**	1.308	0.872	**C9 - N1**	1.234	0.895
**C2 - C3**	0.964	1.092	**C10 - C11**	1.063	1.086
**C2 - H4**	0.809	0.971	**C10 - C13**	1.084	1.014
**C2 - H6**	0.056		**C10 - H3**	0.063	
**C2 - N1**	1.046	0.881	**C10 - H13**	0.067	
**C2 - O1**	1.232	1.015	**C10 - H14**	0.078	
**C3 - C4**	0.974	0.887	**C10 - H15**	0.063	
**C3 - H6**	0.805	0.991	**C11 - H17**	0.064	
**C3 - H7**		0.062	**C11 - N2**	1.227	1.014
**C3 - O2**	1.231	1.128	**C11 - O5**	2.233	1.878
**C4 - C5**	0.988	1.073	**C12 - H17**	0.069	
**C4 - H7**	0.791	0.982	**C12 - N1**	1.198	0.979
**C4 - H10**	0.056		**C12 - N2**	1.263	1.066
**C4 - O3**	1.241	1.192	**C12 - O6**	2.185	1.898
**C5 - C8**	1.05	0.86	**C13 - H3**	0.906	0.944
**C5 - H9**	0.067		**C13 - H13**	0.913	0.979
**C5 - H10**	0.827	0.961	**C13 - H15**	0.906	0.948
**C5 - H12**	0.066		**N1 - H4**	0.058	
**C5 - O1**	1.18	0.982	**N1 - H14**	0.065	
**C6 - C7**	1.09	0.802	**N2 - H17**	0.906	0.839
**C6 - H1**	0.063		**O1 - H4**	0.061	
**C6 - H2**	0.092		**O1 - H10**	0.063	
**C6 - H5**	0.07		**O2 - H6**	0.06	
**C6 - H8**	0.064		**O3 - H2**	0.071	
**C6 - O2**	1.283	0.785	**O3 - H7**	0.059	
**C7 - H1**	0.909	0.972	**O4 - H9**	0.067	
**C7 - H5**	0.914	1.01	**O4 - H11**	1.094	0.931
**C7 - H8**	0.907	0.929	**O4 - H12**	0.062	
**C8 - H9**	0.877	0.96	**O4 - H14**		0.064
**C8 - H10**	0.062				
**C8 - H11**	0.07	0.062			
**C8 - H12**	0.872	1.077			
**C8 - O4**	1.292	0.951			

2) Compound **24a**

Energy: −1067.047549 hartrees

Atomic charges

Atom Label	Natural charge	Mulliken charge	Electrostatic charge
**C1**	+0.095	+0.024	+0.044
**C2**	+0.068	−0.303	+0.378
**C3**	+0.081	−0.295	−0.085
**C4**	+0.318	−0.056	+0.074
**C5**	−0.056	−0.137	−0.182
**C6**	+0.092	−0.023	+0.345
**C7**	−0.597	−0.638	−0.903
**C8**	−0.046	−0.423	−0.074
**C9**	+0.847	+0.438	+0.661
**C10**	+0.065	+0.095	−0.265
**C11**	−0.175	+0.009	+0.108
**C12**	+0.674	+0.053	+0.636
**C13**	−0.593	−0.702	−0.814
**H1**	+0.211	+0.184	+0.25
**H2**	+0.195	+0.187	+0.093
**H3**	+0.197	+0.193	+0.148
**H4**	+0.19	+0.198	+0.123
**H5**	+0.203	+0.181	+0.174
**H6**	+0.196	+0.2	+0.098
**H7**	+0.172	+0.168	+0.135
**H8**	+0.231	+0.229	+0.16
**H9**	+0.209	+0.155	+0.226
**H10**	+0.225	+0.187	+0.264
**H11**	+0.21	+0.166	+0.232
**H13**	+0.175	+0.175	+0.11
**H14**	+0.471	+0.259	+0.43
**H15**	+0.224	+0.19	+0.239
**H16**	+0.225	+0.188	+0.238
**H17**	+0.228	+0.143	+0.231
**H18**	+0.429	+0.392	+0.396
**H22**	+0.176	+0.175	+0.082
**N1**	−0.508	+0.009	−0.044
**N2**	−0.651	−0.458	−0.652
**O1**	−0.608	−0.228	−0.386
**O2**	−0.594	+0.057	−0.317
**O3**	−0.588	−0.084	−0.468
**O4**	−0.737	−0.236	−0.593
**O5**	−0.643	−0.333	−0.561
**O6**	−0.612	−0.338	−0.535

Calculated bond orders

Bond	Löwdin	Mulliken	Bond	Löwdin	Mulliken
**C1 - C2**	0.988	0.975	**C8 - H14**	0.07	0.064
**C1 - C8**	1.051	0.897	**C8 - O4**	1.292	0.978
**C1 - H2**	0.827	0.956	**C9 - H18**	0.069	
**C1 - H3**	0.066		**C9 - N1**	1.2	0.985
**C1 - H13**	0.067		**C9 - N2**	1.261	1.065
**C1 - O1**	1.176	0.91	**C9 - O5**	2.185	1.904
**C2 - C3**	0.978	1.008	**C10 - C11**	1.672	1.792
**C2 - H2**	0.056		**C10 - H17**	0.803	0.91
**C2 - H6**	0.789	0.951	**C10 - N1**	1.239	0.927
**C2 - O3**	1.262	1.185	**C11 - C12**	1.065	1.109
**C3 - C4**	0.964	1.056	**C11 - C13**	1.084	1.025
**C3 - H4**	0.802	0.97	**C11 - H9**	0.067	
**C3 - O2**	1.26	1.085	**C11 - H15**	0.062	
**C4 - H4**	0.057		**C11 - H16**	0.063	
**C4 - H8**	0.81	0.962	**C11 - H17**	0.077	
**C4 - N1**	1.041	0.872	**C12 - H18**	0.064	
**C4 - O1**	1.232	1.051	**C12 - N2**	1.228	1.008
**C5 - C6**	1.03	0.892	**C12 - O6**	2.23	1.873
**C5 - H5**	0.874	0.994	**C13 - H9**	0.913	0.962
**C5 - H7**	0.858	0.966	**C13 - H15**	0.907	0.962
**C5 - H22**	0.056		**C13 - H16**	0.906	0.943
**C5 - O3**	1.243	0.906	**N1 - H8**	0.058	
**C6 - C7**	1.091	0.994	**N1 - H17**	0.065	
**C6 - H1**	0.061		**N2 - H18**	0.907	0.84
**C6 - H5**	0.062		**O1 - H2**	0.062	
**C6 - H10**	0.065		**O1 - H8**	0.061	
**C6 - H11**	0.07		**O2 - H4**	0.061	
**C6 - H22**	0.834	0.988	**O2 - H22**	0.064	
**C6 - O2**	1.204	0.893	**O3 - H5**	0.064	
**C7 - H1**	0.912	0.937	**O3 - H6**	0.059	
**C7 - H10**	0.915	0.962	**O3 - H7**	0.064	
**C7 - H11**	0.917	1.02	**O4 - H3**	0.062	
**C8 - H2**	0.063		**O4 - H13**	0.067	
**C8 - H3**	0.872	1.075	**O4 - H14**	1.095	0.933
**C8 - H13**	0.877	0.96	**O4 - H17**		0.067

3) Compound **28a**

Energy: −1106.366651 hartrees

Atomic charges

Atom Label	Natural charge	Mulliken charge	Electrostatic charge
**C1**	+0.317	−0.231	−0.001
**C2**	+0.096	+0.063	−0.05
**C3**	+0.068	−0.419	+0.513
**C4**	+0.083	−0.226	+0.048
**C5**	+0.126	+0.06	+0.605
**C6**	−0.457	−0.313	−0.571
**C7**	−0.023	−0.245	−0.002
**C8**	−0.586	−0.653	−0.915
**C9**	−0.045	−0.404	−0.045
**C10**	+0.851	+0.481	+0.674
**C11**	+0.066	+0.038	−0.313
**C12**	−0.175	+0.028	+0.156
**C13**	+0.674	+0.03	+0.612
**C14**	−0.593	−0.659	−0.832
**H1**	+0.163	+0.166	+0.082
**H2**	+0.196	+0.195	+0.107
**H3**	+0.209	+0.154	+0.228
**H4**	+0.231	+0.242	+0.169
**H5**	+0.196	+0.192	+0.139
**H6**	+0.197	+0.19	+0.043
**H7**	+0.204	+0.165	+0.226
**H8**	+0.22	+0.178	+0.247
**H9**	+0.165	+0.173	+0.006
**H10**	+0.195	+0.218	+0.104
**H11**	+0.224	+0.179	+0.18
**H12**	+0.216	+0.202	+0.208
**H13**	+0.208	+0.182	+0.241
**H14**	+0.196	+0.178	+0.148
**H15**	+0.175	+0.175	+0.109
**H16**	+0.471	+0.261	+0.435
**H17**	+0.225	+0.194	+0.245
**H18**	+0.224	+0.147	+0.236
**H19**	+0.224	+0.19	+0.237
**H20**	+0.429	+0.393	+0.395
**N1**	−0.503	−0.02	−0.034
**N2**	−0.652	−0.461	−0.649
**O1**	−0.608	−0.206	−0.35
**O2**	−0.599	−0.064	−0.522
**O3**	−0.613	+0.129	−0.412
**O4**	−0.739	−0.238	−0.605
**O5**	−0.642	−0.328	−0.564
**O6**	−0.613	−0.335	−0.53

Calculated bond orders

Bond	Löwdin	Mulliken	Bond	Löwdin	Mulliken
**C1 - C4**	0.949	1.092	**C8 - H13**	0.911	0.929
**C1 - H4**	0.809	0.978	**C9 - H2**	0.061	
**C1 - H10**	0.055		**C9 - H5**	0.871	1.087
**C1 - N1**	1.047	0.864	**C9 - H15**	0.877	0.952
**C1 - O1**	1.238	1.067	**C9 - H16**	0.071	0.068
**C2 - C3**	0.978	1.016	**C9 - O4**	1.291	0.952
**C2 - C9**	1.047	0.812	**C10 - H20**	0.068	
**C2 - H2**	0.822	0.953	**C10 - N1**	1.199	0.971
**C2 - H5**	0.065		**C10 - N2**	1.257	1.064
**C2 - H15**	0.067		**C10 - O5**	2.188	1.913
**C2 - O1**	1.203	0.889	**C11 - C12**	1.672	1.766
**C3 - C4**	0.976	0.969	**C11 - H18**	0.802	0.917
**C3 - H2**	0.056		**C11 - N1**	1.234	0.96
**C3 - H6**	0.792	0.937	**C12 - C13**	1.062	1.098
**C3 - H10**	0.056		**C12 - C14**	1.084	0.981
**C3 - O2**	1.263	1.322	**C12 - H3**	0.067	
**C4 - H6**		0.074	**C12 - H17**	0.062	
**C4 - H10**	0.809	0.975	**C12 - H18**	0.078	
**C4 - O3**	1.255	1.017	**C12 - H19**	0.063	
**C5 - C6**	1.039	0.947	**C13 - H20**	0.064	
**C5 - C8**	1.087	1.016	**C13 - N2**	1.232	1.026
**C5 - H7**	0.069		**C13 - O6**	2.229	1.861
**C5 - H8**	0.065		**C14 - H3**	0.913	0.947
**C5 - H9**	0.826	0.901	**C14 - H17**	0.906	0.987
**C5 - H11**	0.063		**C14 - H19**	0.906	0.938
**C5 - H12**	0.056		**N1 - H4**	0.058	
**C5 - H13**	0.061	0.056	**N1 - H18**	0.065	
**C5 - O3**	1.206	0.932	**N2 - H20**	0.907	0.839
**C6 - C7**	1.073	0.906	**O1 - H2**	0.064	
**C6 - H1**	0.057		**O1 - H4**	0.062	
**C6 - H11**	0.868	0.956	**O2 - H1**	0.066	
**C6 - H12**	0.862	1.012	**O2 - H6**	0.058	
**C6 - H14**	0.061		**O2 - H14**	0.069	
**C7 - H1**	0.862	0.946	**O3 - H9**	0.066	
**C7 - H11**	0.069		**O3 - H10**	0.066	
**C7 - H14**	0.878	1.007	**O4 - H5**	0.062	
**C7 - O2**	1.258	0.918	**O4 - H15**	0.067	
**C8 - H7**	0.919	1.007	**O4 - H16**	1.093	0.929
**C8 - H8**	0.911	0.999	**O4 - H18**		0.057
**C8 - H9**		0.068	**O4 - H18**		0.057

4) Compound **29a**

Energy: −1106.366312 hartrees

Atomic charges

Atom Label	Natural charge	Mulliken charge	Electrostatic charge
**C1**	+0.319	−0.127	+0.012
**C2**	+0.097	−0.099	−0.089
**C3**	+0.074	−0.322	+0.672
**C4**	+0.079	−0.284	−0.136
**C5**	+0.13	+0.057	+0.538
**C6**	−0.459	−0.171	−0.575
**C7**	−0.024	−0.306	+0.005
**C8**	−0.592	−0.755	−0.911
**C9**	−0.045	−0.349	−0.048
**C10**	+0.85	+0.474	+0.655
**C11**	+0.075	+0.061	−0.269
**C12**	−0.176	+0.002	+0.14
**C13**	+0.675	+0.056	+0.603
**C14**	−0.592	−0.673	−0.818
**H1**	+0.193	+0.176	+0.147
**H2**	+0.193	+0.201	+0.11
**H3**	+0.209	+0.155	+0.225
**H4**	+0.23	+0.235	+0.179
**H5**	+0.198	+0.193	+0.14
**H6**	+0.196	+0.217	+0.031
**H7**	+0.221	+0.188	+0.248
**H8**	+0.206	+0.165	+0.231
**H9**	+0.209	+0.191	+0.246
**H10**	+0.193	+0.198	+0.134
**H11**	+0.216	+0.202	+0.203
**H12**	+0.226	+0.178	+0.185
**H14**	+0.164	+0.167	+0.082
**H15**	+0.174	+0.172	+0.111
**H16**	+0.47	+0.26	+0.43
**H17**	+0.224	+0.191	+0.237
**H18**	+0.225	+0.149	+0.229
**H19**	+0.224	+0.188	+0.239
**H20**	+0.428	+0.394	+0.384
**H25**	+0.167	+0.171	+0.038
**N1**	−0.503	−0.082	−0.041
**N2**	−0.651	−0.469	−0.612
**O1**	−0.609	−0.18	−0.341
**O2**	−0.605	−0.096	−0.563
**O3**	−0.61	+0.18	−0.351
**O4**	−0.738	−0.235	−0.6
**O5**	−0.647	−0.337	−0.568
**O6**	−0.613	−0.336	−0.53

Calculated bond orders

Bond	Löwdin	Mulliken	Bond	Löwdin	Mulliken
**C1 - C4**	0.946	1.065	**C9 - H5**	0.871	1.087
**C1 - H4**	0.809	0.969	**C9 - H15**	0.877	0.946
**C1 - N1**	1.048	0.909	**C9 - H16**	0.071	0.069
**C1 - O1**	1.241	1.07	**C9 - O4**	1.291	0.942
**C2 - C3**	0.98	1.068	**C10 - H20**	0.069	
**C2 - C9**	1.047	0.85	**C10 - N1**	1.202	0.971
**C2 - H2**	0.823	0.962	**C10 - N2**	1.26	1.067
**C2 - H5**	0.065		**C10 - O5**	2.182	1.903
**C2 - H15**	0.067		**C11 - C12**	1.673	1.775
**C2 - O1**	1.199	0.903	**C11 - H18**	0.803	0.919
**C3 - C4**	0.977	0.999	**C11 - N1**	1.231	0.972
**C3 - H2**	0.056		**C12 - C13**	1.064	1.116
**C3 - H6**	0.794	0.941	**C12 - C14**	1.083	1.019
**C3 - O2**	1.262	1.234	**C12 - H3**	0.067	
**C4 - H10**	0.807	0.938	**C12 - H17**	0.063	
**C4 - O3**	1.253	1.012	**C12 - H18**	0.077	
**C5 - C6**	1.041	0.949	**C12 - H19**	0.063	
**C5 - C8**	1.086	0.958	**C13 - H20**	0.064	
**C5 - H7**	0.064		**C13 - N2**	1.228	1.03
**C5 - H8**	0.068		**C13 - O6**	2.229	1.864
**C5 - H9**	0.06	0.06	**C14 - H3**	0.913	0.956
**C5 - H12**	0.065		**C14 - H17**	0.907	0.976
**C5 - H25**	0.83	0.962	**C14 - H19**	0.906	0.934
**C5 - O3**	1.213	0.852	**N1 - H4**	0.058	
**C6 - C7**	1.07	0.852	**N1 - H18**	0.065	
**C6 - H1**	0.062		**N2 - H20**	0.907	0.838
**C6 - H11**	0.862	1.028	**O1 - H2**	0.064	
**C6 - H12**	0.869	0.954	**O1 - H4**	0.063	
**C6 - H14**	0.057		**O2 - H1**	0.071	
**C6 - H25**	0.056		**O2 - H6**	0.062	
**C7 - H1**	0.879	1.023	**O2 - H14**	0.07	
**C7 - H12**	0.069		**O3 - H10**	0.061	
**C7 - H14**	0.865	0.969	**O3 - H25**	0.065	
**C7 - O2**	1.248	0.804	**O4 - H5**	0.062	
**C8 - H7**	0.914	0.976	**O4 - H15**	0.067	
**C8 - H8**	0.919	1.022	**O4 - H16**	1.094	0.931
**C8 - H9**	0.91	0.948	**O4 - H18**		0.057
**C9 - H2**	0.061				
